# Determinants of Isoform-Specific Gating Kinetics of hERG1 Channel: Combined Experimental and Simulation Study

**DOI:** 10.3389/fphys.2018.00207

**Published:** 2018-04-12

**Authors:** Laura L. Perissinotti, Pablo M. De Biase, Jiqing Guo, Pei-Chi Yang, Miranda C. Lee, Colleen E. Clancy, Henry J. Duff, Sergei Y. Noskov

**Affiliations:** ^1^Centre for Molecular Simulations, Department of Biological Sciences, Faculty of Science, University of Calgary, Calgary, AB, Canada; ^2^Libin Cardiovascular Institute of Alberta, Faculty of Medicine, University of Calgary, Calgary, AB, Canada; ^3^Department of Physiology and Membrane Biology, University of California, Davis, Davis, CA, United States

**Keywords:** long-QT, hERG Isoforms, gating kinetics, arrhythmias, computational models, Markov process

## Abstract

I_Kr_ is the rapidly activating component of the delayed rectifier potassium current, the ion current largely responsible for the repolarization of the cardiac action potential. Inherited forms of long QT syndrome (LQTS) (Lees-Miller et al., 1997) in humans are linked to functional modifications in the Kv11.1 (hERG) ion channel and potentially life threatening arrhythmias. There is little doubt now that hERG-related component of I_Kr_ in the heart depends on the tetrameric (homo- or hetero-) channels formed by two alternatively processed isoforms of hERG, termed hERG1a and hERG1b. Isoform composition (hERG1a- vs. the b-isoform) has recently been reported to alter pharmacologic responses to some hERG blockers and was proposed to be an essential factor pre-disposing patients for drug-induced QT prolongation. Very little is known about the gating and pharmacological properties of two isoforms in heart membranes. For example, how gating mechanisms of the hERG1a channels differ from that of hERG1b is still unknown. The mechanisms by which hERG 1a/1b hetero-tetramers contribute to function in the heart, or what role hERG1b might play in disease are all questions to be answered. Structurally, the two isoforms differ only in the N-terminal region located in the cytoplasm: hERG1b is 340 residues shorter than hERG1a and the initial 36 residues of hERG1b are unique to this isoform. In this study, we combined electrophysiological measurements for HEK cells, kinetics and structural modeling to tease out the individual contributions of each isoform to Action Potential formation and then make predictions about the effects of having various mixture ratios of the two isoforms. By coupling electrophysiological data with computational kinetic modeling, two proposed mechanisms of hERG gating in two homo-tetramers were examined. Sets of data from various experimental stimulation protocols (HEK cells) were analyzed simultaneously and fitted to Markov-chain models (M-models). The minimization procedure presented here, allowed assessment of suitability of different Markov model topologies and the corresponding parameters that describe the channel kinetics. The kinetics modeling pointed to key differences in the gating kinetics that were linked to the full channel structure. Interactions between soluble domains and the transmembrane part of the channel appeared to be critical determinants of the gating kinetics. The structures of the full channel in the open and closed states were compared for the first time using the recent Cryo-EM resolved structure for full open hERG channel and an homology model for the closed state, based on the highly homolog EAG1 channel. Key potential interactions which emphasize the importance of electrostatic interactions between N-PAS cap, S4-S5, and C-linker are suggested based on the structural analysis. The derived kinetic parameters were later used in higher order models of cells and tissue to track down the effect of varying the ratios of hERG1a and hERG1b on cardiac action potentials and computed electrocardiograms. Simulations suggest that the recovery from inactivation of hERG1b may contribute to its physiologic role of this isoform in the action potential. Finally, the results presented here contribute to the growing body of evidence that hERG1b significantly affects the generation of the cardiac I_kr_ and plays an important role in cardiac electrophysiology. We highlight the importance of carefully revisiting the Markov models previously proposed in order to properly account for the relative abundance of the hERG1 a- and b- isoforms.

## Introduction

**Figure 1:**
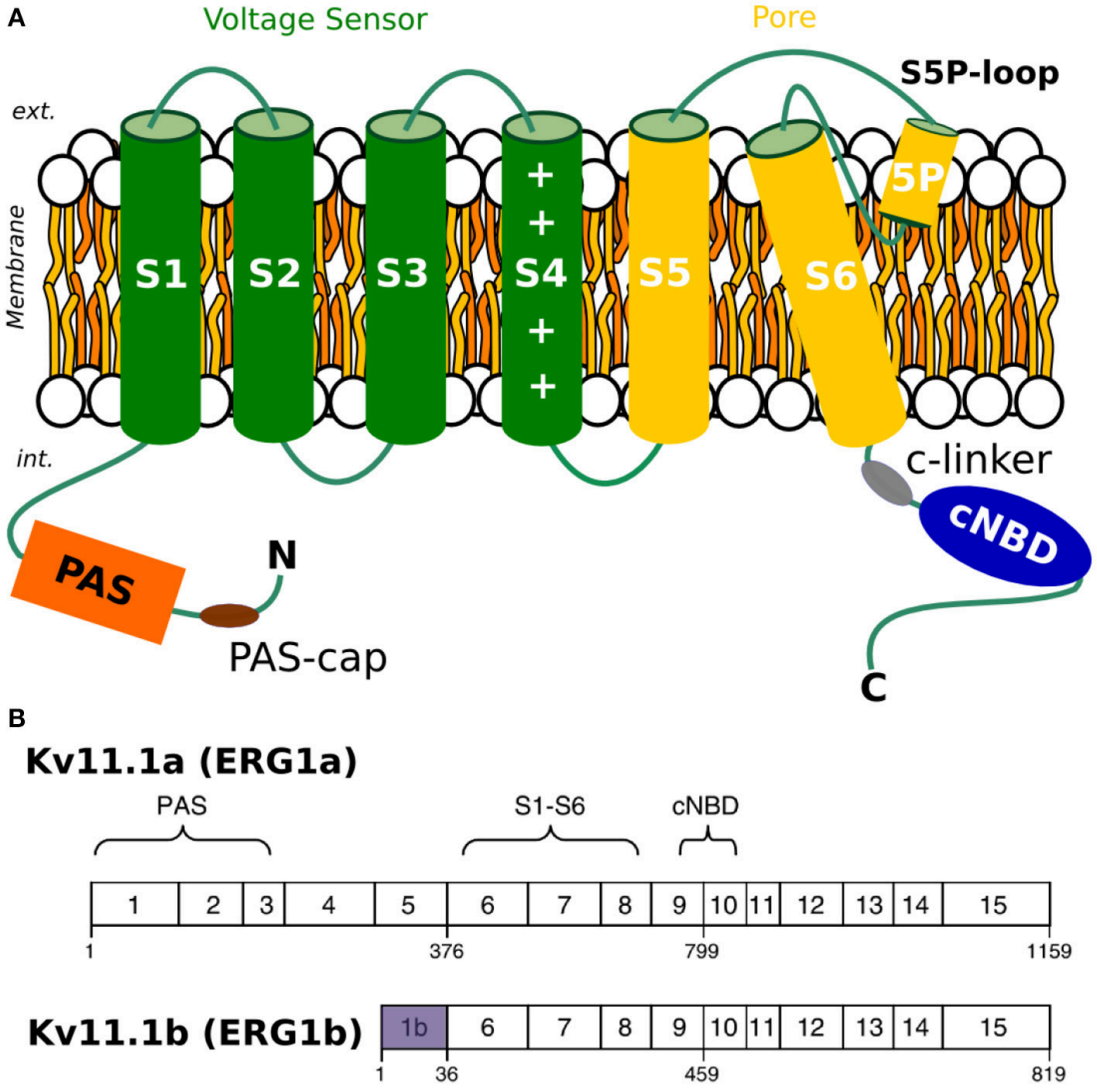
**(A)** Topology of Kv11.1 or hERG channel. **(B)** Comparison of a-isoform and b-isoform. Exons of each of the two splice variants, with a-isoform on the top and b-isoform on the bottom.

The I_Kr_ current is a primary contributor to the repolarization of the human cardiac muscle, a delayed rectifier potassium current conducted by the Kv11.1 ion channel (more commonly referred to as human ether-a-go-go-related gene, or hERG1) (Sanguinetti et al., 1995; Li et al., 1996). The Kv11.1 channel is homologous in structure to other voltage-gated potassium channels (Figure 1) and is assembled as a tetramer to become a fully functioning ion channel, but has very different kinetics compared to other potassium channels. Inactivation is much faster than activation, and consequently, current is suppressed at positive potentials but rebounds on repolarization as channels quickly recover from inactivation and slowly close. During an action potential, this gating behavior produces a resurgent current that peaks during the repolarization phase. Mutations, channels block by drugs and/or impaired trafficking of Kv11.1 channels to the cell membrane lead to prolongation of the QT interval on the surface electrocardiogram (LQTS), leading to a potentially life threatening ventricular arrhythmia (Behere et al., 2014). Since the physiological role of I_Kr_ is to repolarize the late phase of cardiac action potentials, hERG1 has a clear link to these arrhythmias (Robertson et al., 2008; Gustina and Trudeau, 2009; Robertson, 2012; Vandenberg et al., 2012). That is, if I_Kr_ is reduced, due to loss-of-function mutations or action of small molecules (drugs), patients are more likely to develop severe arrhythmias initiated by premature beats.

Up to date, our understanding of how I_Kr_ contributes to the ventricular repolarization is based primarily on studies utilizing heterologous expression of the originally identified hERG1 a-isoform (Sanguinetti et al., 1995; Trudeau et al., 1995; Smith et al., 1996; Wang et al., 1997). More recent studies showed that native I_Kr_ result from hetero-tetramers formed by the co-assembly of two hERG isoforms termed hERG1a and hERG1b. Two splice variants—hERG1a and hERG1b are co-expressed not only in cardiac tissue, but also in neurons and smooth muscles (Chiesa et al., 1997; Ohya et al., 2002). Importantly, isoforms display very different gating kinetics (Lees-Miller et al., 2003). The hERG gating is modulated by the cytoplasmic domains (N-terminal or PAS domain, CNBD and C-linker) in a way that still remains largely unknown but of a critical importance for unraveling structural mechanisms responsible for QT prolongation. In particular, a mutation in the N-terminal of hERG1b was discovered in a patient with long QT Syndrome (LQTS), highlighting the importance of this isoform in cardiac repolarization (Robertson et al., 2008; Robertson, 2012).

Many drugs are known to block ion current across Kv11.1 channels, resulting in an acquired form of LQTS (Larsen et al., 2010). Many blockers exhibit state-dependent activity and hence their propensity to later hERG currents is related to the channel's gating kinetics. It has recently been shown that EA4031, a selective blocker of hERG1 currents, differs in effectiveness on homo-tetrameric vs. hetero-tetrameric channels formed of different isoforms (Sale et al., 2008). Similar findings were also reported for hERG1 activators. Larsen et al. (2010) showed that activators such as NS1643 display differential effects on the homo-tetrameric channels formed by two hERG1 isoforms (Holzem et al., 2016). Due to the therapeutic risks hidden in hERG1 blockers and potential of hERG1 activators, establishing differences in gating mechanisms of two isoforms is critically important. Structurally, the two isoforms differ only in the N-terminal region located in the cytoplasm: hERG1b is 340 residues shorter than hERG1a and the initial 36 residues of hERG1b are unique to this isoform (Lees-Miller et al., 1997; Splawski et al., 1998) (Figure 1). As mentioned above, the channel gating is modulated by the cytoplasmic domains (PAS, CNBD, and C-linker) in a way that still remains unknown (Trudeau et al., 2011; Ng et al., 2014; Morais-Cabral and Robertson, 2015; Perry et al., 2015). Consequently, as hERG1b is lacking the entire PAS/Pas-cap domains, it has a different gating behavior compared to hERG1a.

The isoform originally discovered was hERG1a which is considered the full length transcript of the associated gene, and is often referred to simply as hERG1 when not being compared to other isoforms. Additionally, these isoforms are present in relatively fixed ratios, which depend on the cellular environment (Larsen et al., 2007, 2008). Deviations from these ratios, leading to abnormal abundance of a particular Kv11.1 isoform, may result in heart beat anomalies (Larsen et al., 2008; Kannankeril et al., 2010; Robertson, 2012).

Recently, hERG1b was found to be critical for human cardiac repolarization and a 1b-specific mutation associated with intrauterine fetal death was discovered (Jones et al., 2014, 2015, 2016). Additionally, the relative levels of expression appear to be greater in the young compared with the adult heart (Wang et al., 2008; Crotti et al., 2013). Evidence supports that when hERG1a and hERG1b are present in heterologous expression systems, they co-assemble to form hetero-tetrameric channels, although it is unknown if there is a preferred stoichiometry of these channels (London et al., 1997).

As previously mentioned, the two isoforms gating properties differ substantially. The hERG1 b- isoform is characterized by faster kinetics of activation, recovery from inactivation, and most prominently, deactivation (Larsen et al., 2008, 2010). These differences in gating kinetics are due mainly to the differences in the N-terminal regions of the two isoforms. More specifically, steady state activation is affected by the absence of the proximal N-terminal region in hERG1b, and the activation rate is suggested to be dependent on a short sequence of residues in the proximal portion of the hERG1a N-terminus (Saenen et al., 2006; Trudeau et al., 2011). Consequently, activation rates are much faster in hERG1b channels where these residues are missing (Larsen et al., 2008). Regarding deactivation, it has been suggested that the slow deactivation of hERG1a channels might be facilitated by the first 16 residues of the N-terminus, among other factors (Wang et al., 2008). According to that, faster deactivation rates in hERG1b can be explained by the presence of a unique N-terminal. The inactivation rate was shown to be similar between the two isoforms (Larsen et al., 2008). This finding is expected, as the mechanism by which fast inactivation occurs has been proposed to rely mainly on voltage induced changes in the structure of the outer mouth of the pore (Schönherr and Heinemann, 1996; Perry et al., 2013a,b; Thomson et al., 2014) and the sequence spanning this region is identical in both isoforms. Lastly, recovery from inactivation is significantly faster in hERG1b compared to hERG1a, potentially implying that by some means, the N-terminus contributes to this process with already proposed stabilizing interactions (Saenen et al., 2006; Gustina and Trudeau, 2011).

**Figure 2:**
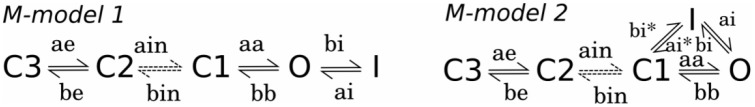
Proposed kinetic mechanisms using Markov models (M-models) for WT hERG (Perissinotti et al., 2015; Romero et al., 2015) showing the transition rates with the corresponding labels.

Despite the evidence that heterometric hERG 1a/1b channels underlie cardiac I_kr_, little is known about the gating and pharmacological properties of these channels, how hERG1a channels differ from hERG1b homomers, 1a/1b heteromers, or which role hERG1b might play in disease. Two broadly accepted gating mechanisms were established on the basis of kinetic modeling driven by the experimental data from electrophysiology studies of Kv11.1a channel. The first gating mechanism that successfully describes gating kinetics was proposed by Rasmusson, and later refined by Fink et al. and Romero et al. (termed M-model 1, Figure 2) (Wang et al., 1997; Fink et al., 2008; Romero et al., 2015). The modified M-model 1 (Fink et al., 2008) for hERG1 channel has been combined with the cardiac cell model (Ten-Tusscher Model; Ten Tusscher and Panfilov, 2006) in order to reproduce, and explain in terms of kinetics, measurements in oocytes, and HEK cells and showed overall good performance. The second hERG1 current scheme with different connectivity between gating states was developed by both Clancy et al. (Clancy and Rudy, 2001; Clancy et al., 2007) and Mazhari et al. (2001) (termed M-model2, Figure 2) on the basis of M-model originally proposed by Kiehn et al. (1999).

There are only a limited number of studies that employ kinetic modeling to understanding of gating kinetics in hERG1 isoforms. Sale et al. (2008) previously attempted to study the hetero channels formed by hERG1a/1b in HEK cells in presence and absence of E-4031 blocker. The M-model 2 was used to describe gating process in a-isoform and a-,b- heteromer. To explain apparent challenges in fitting experimental currents, Sale et al. proposed that the presence of the extended N termini in all 4 subunits in hERG 1a may alter gating process; hence an alternative gating mode (“N-mode”) was considered. Another previous work modified the M-model 1 parameters proposed by Fink et al. (2008) and implemented them in the cardiac cell model (Ten Tusscher and Panfilov, 2006) in order to reproduce, and explain measurements in oocytes and HEK cells. However, none of the previous works (Robertson et al., 2008; Sale et al., 2008; Larsen et al., 2010; Holzem et al., 2016) tested the quality of the proposed kinetic models in fitting the hERG1b homo-tetramer experimental data, nor attempted to derive the set of model parameters for this isoform or suggest structural mechanisms explaining differences in isoform gating kinetics (Wacker et al., 2017).

The rapid progress in the structural biology finally resulted in the Cryo-EM high-resolution structure of hERG1 channel. The structure of the full hERG1 channel in its open state together with other highly homologous channels EAG1 and other closely-related channels from CNG and HCN families were published in 2016-2017 (Whicher and Mackinnon, 2016; Lee and Mackinnon, 2017; Li et al., 2017; Wang and Mackinnon, 2017). The availability of this new structural data provides a unique opportunity to connect well-established kinetic models of hERG1 channel to its structural determinant.

This work is striving to achieve several goals. First goal is the methodological one, where we developed and compared optimal gating schemes for hERG a- and b- isoforms. The second goal is to provide a perspective view on the potential structural mechanisms responsible for apparent kinetic differences between two isoforms and then to explore and discuss its implications at the tissue level. To achieve our methodological goal we systematically compared two gating schemes using available and novel electrophysiological recordings performed in HEK cell lines. The kinetic schemes illuminated profound differences in deactivation kinetics between two isoforms. To understand underlying reasons for different deactivation process, we employed structural modeling of hERG1 channel in open and closed-states using recently published structures of EAG1 and hERG1 channels from Cryo-EM (Whicher and Mackinnon, 2016; Wang and Mackinnon, 2017). We found that the available structures allowed identification of potential mutants with altered kinetics in good agreement with developed kinetic models. Finally, to provide a perspective on the potential role of isoforms in cellular dynamics, we undertook the cardiac cell simulations to reveal the conditions (i.e., isoform composition of hERG channels) leading to QT alterations. To provide initial glimpses into cellular roles of different isoform expression, a selected kinetic model, together with the optimized parameters, was incorporated into a higher dimensional model of the cardiac cell (O'hara et al., 2011) to simulate cellular and tissue dynamics effects as function of hERG isoform ratio.

## Materials and methods

### Expression of hERG1a and hERG1b in HEK cells

Lees-Miller et al. first reported the electrophysiology of the hERG1 b-isoform (Lees-Miller et al., 1997). The hERG1 b-isoform was cloned from human atrium. hERG1 isoforms were cloned into the pIRES-hr green fluorescent protein-1a vector (Agilent Technologies, Santa Clara, CA) for co-expression with humanized Renilla reniformis GPF. Human embryonic kidney (HEK) 293 cells were transfected by using calcium phosphate and cultured in Dulbecco's modified Eagle's medium supplemented with 10% horse serum (Invitrogen, Carlsbad, CA). Transfection was monitored by green fluorescence. HEK cells were chosen because their background potassium currents are small. More importantly, no dofetilide-sensitive tail current has been observed by using the voltage-clamp protocol in un-transfected HEK cells.

#### General setup for electrophysiological recordings

Transfected HEK cells on glass coverslips were placed in a chamber mounted on a modified stage of an inverted microscope. The chamber was superfused at a rate of 2 ml/min with a normal external solution. Micropipettes were pulled from borosilicate glass capillary tubes on a programmable horizontal puller (Sutter Instrument Company, Novato, CA). Standard patch-clamp methods were used to measure the whole-cell currents of hERG1 mutants expressed in HEK 293 cells by using the Axopatch 200B amplifier (Molecular Devices, Sunnyvale, CA) (Lees-Miller et al., 2009). The pipette solution contained the following: 10 mM KCl, 110 mM K-aspartate, 5 mM MgCl_2_, 5 mM Na_2_ATP, 10 mM ethylene glycol-bis(β-aminoethyl ether)-N,N,N′,N′tetraacetic acid, 5 mM HEPES, and 1 mM CaCl_2_. The solution was adjusted to pH 7.2 with KOH. The EC solution contained the following: 140 mM NaCl, 5.4 mM KCl, 1 mM CaCl_2_, 1 mM MgCl_2_, 5 mM HEPES, and 5.5 mM glucose. The solution was adjusted to pH 7.4 with NaOH. In patch clamp experiments, serious resistance and capacitance during the whole cell patch clamp recording were compensated to 90% through Axopatch 200B patch clamp amplifier. Whole cell patch clamp experiments were performed when access resistance was <10 MOme. No leak subtraction was performed. The junction potential of −10 mV was adjusted on all the membrane potentials recorded. All experiments were conducted at room temperature.

#### Voltage protocols

##### Voltage-dependence of activation

**Figure 3:**
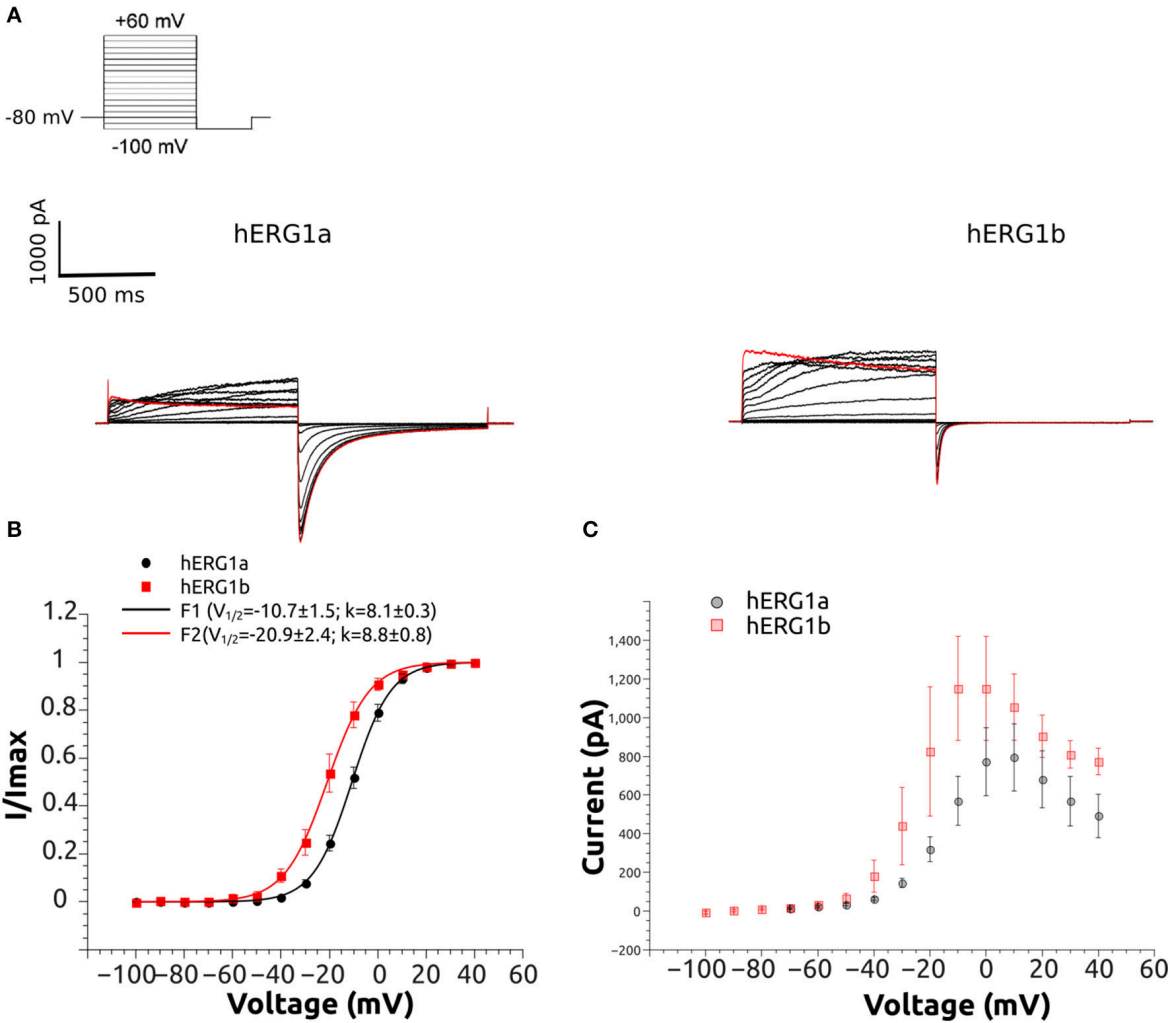
Representative current traces of hERG1a and hERG1b channels and their current-voltage relationship at room temperature. **(A)** Selected current traces recorded from HEK cells and elicited by the voltage protocol shown in the top. **(B)** Normalized tail currents against voltage. The solid lines correspond to the fitted Boltzmann functions and symbols correspond to data. All data are listed as mean ± SEM. hERG1a (*n* = 10), hERG1b (*n* = 10). **(C)** Currents measured at the end of each step were used to construct the current-voltage (I-V) relationship. All data are shown as mean ± SEM. hERG1a (*n* = 10), hERG1b (*n* = 10).

From a holding potential of −80 mV cells were depolarized for 1 s to a range of voltages from −100 to +40 mV followed by a step to −100 mV (1 s) to record the tail currents (Figure 3). The isochronal tail current-voltage plots were fit to a single Boltzmann function (1):

(1) $$\begin{array}{r} \frac{I}{I_{max}}=\frac{1}{\left(1+exp\left[\frac{\left(V_{\frac{1}{2}}-V_{m}\right)}{k}\right]\right)} \end{array}$$

Where *I / I*_*max*_ is the normalized current, *V*_1/2_ is voltage of the half-maximal activation, *k* is the slope factor and V_m_ is the membrane potential.

##### Envelope of tails

**Figure 4:**
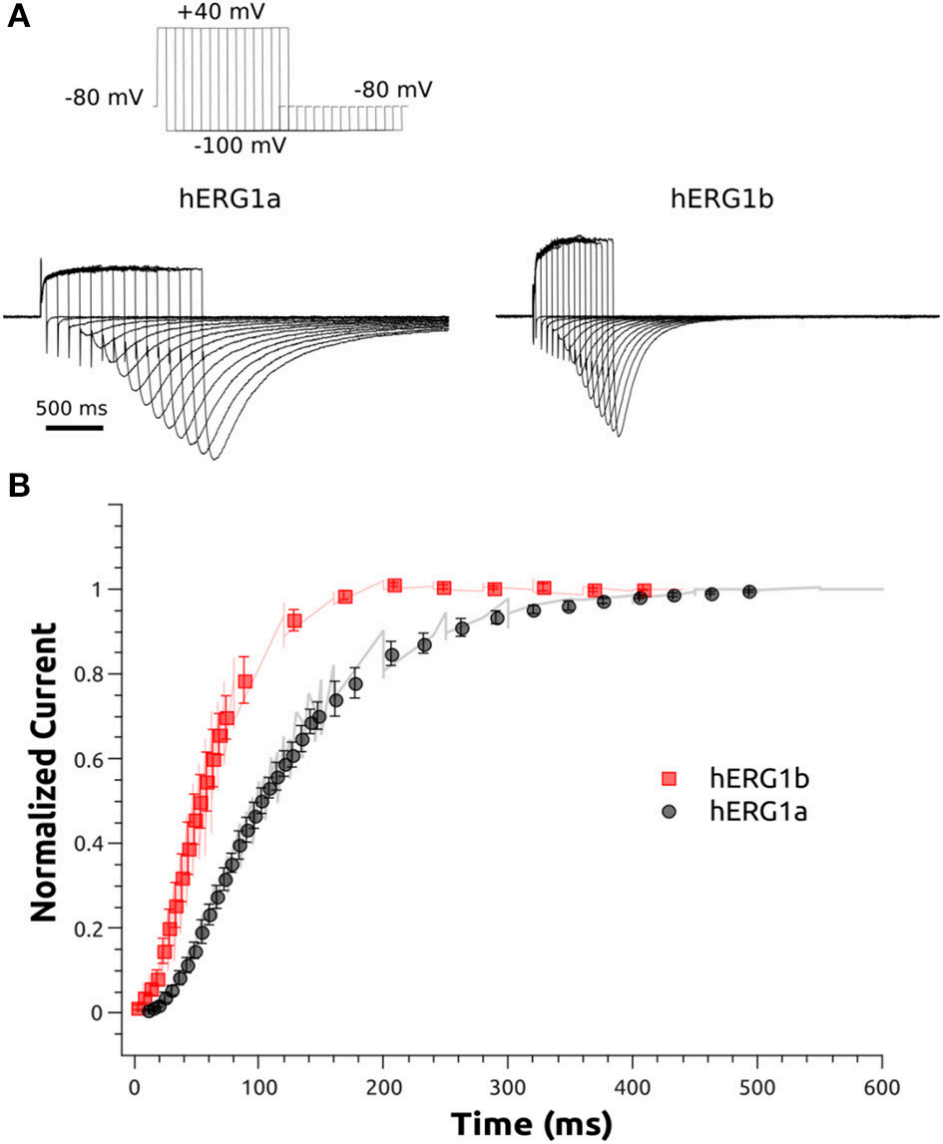
Activation kinetics of hERG1a and hERG1b channels expressed in HEK cells. An envelope of tails protocol was used to measure the activation properties at +40 mV. **(A)** Representative current traces elicited by the protocol shown at the top corresponding to 5–500 ms of activation are shown. **(B)**The data were normalized to the maximum amplitude of the tail current and plotted against time. hERG1a (*n* = 10); hERG1b (*n* = 10). All data are shown as mean ± SEM.

The activation of hERG1a and hERG1b channels was examined at +40 mV in HEK cells. The protocol is shown in Figure 4. The measurements were carried out by activating the channels at +40 mV for various durations of time (from 5 to 500 ms) and then measuring the tail current at −100 mV (3 s). The peak amplitude of the tail current was used as a measure of the relative amount of activated channels at a given time point. The peak amplitudes were normalized to the maximum amplitude and plotted as a function of the duration of the activating step.

##### Deactivation

**Figure 5:**
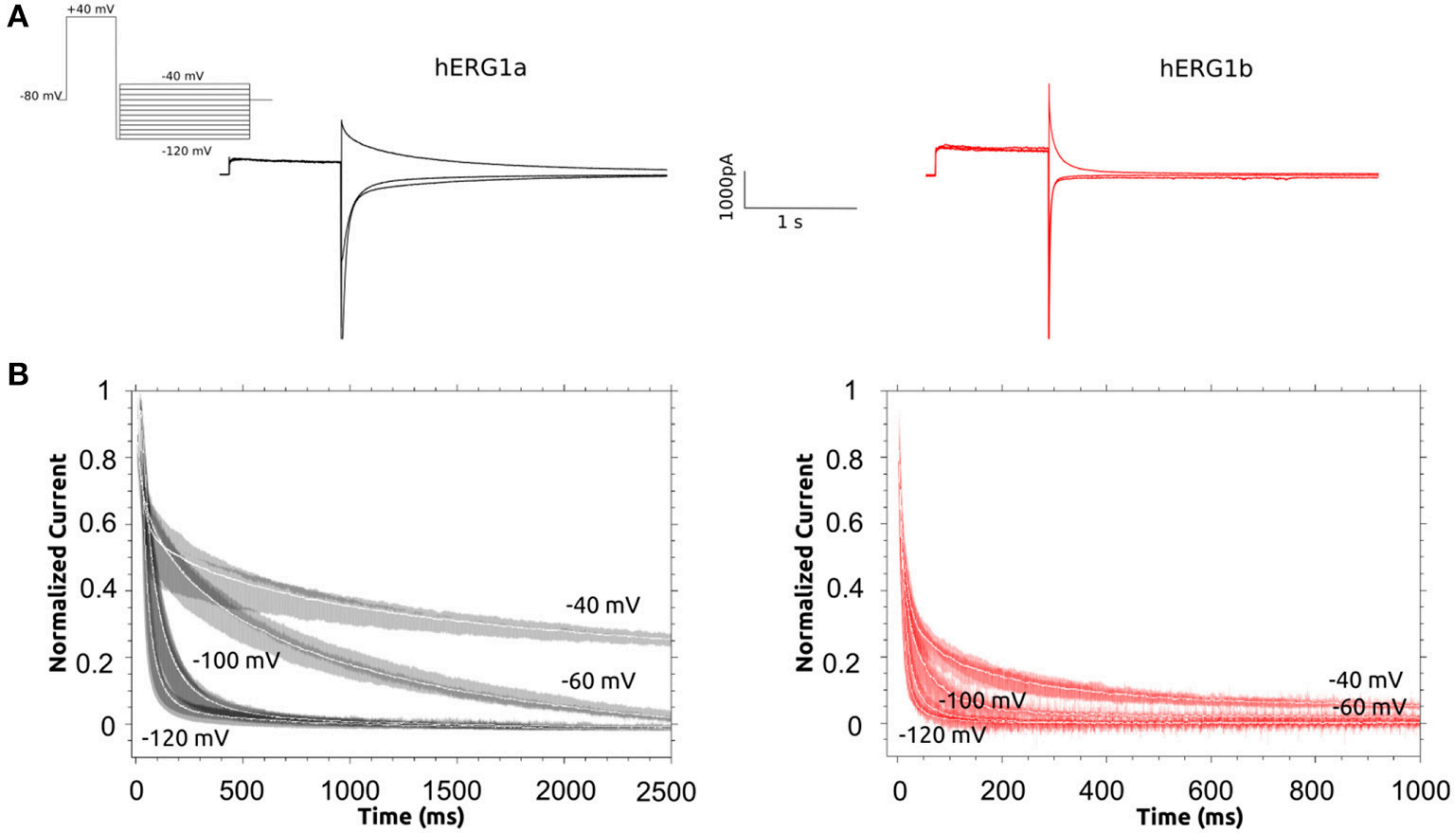
Deactivation Kinetics of hERG1a and hERG1b channels measured in HEK cells. **(A)** Representative current traces elicited by the voltage protocol shown in the top for both isoforms. **(B)** Superimposed raw deactivation time courses for voltages −40, −60, −100, and −120 mV for hERG1a (*n* = 10) and hERG1b (*n* = 10). Average time course is shown with a solid white line.

Deactivation of hERG1a/1b tail current was measured by activating channels al +40, followed with a short (5 ms) repolarization step to −120 mV and deactivating step at −120, −100, −60, −40 mV. Currents at different voltages were normalized and averaged (*n* = 10) time course data was plotted for each isoform at the different voltages (Figure 5).

##### Statistical analysis

Statsview (Abacus Concepts, Berkeley, CA) or QTIplot (Vasilef, 2013), Grace (http://plasma-gate.weizmann.ac.il/Grace/) were used to analyze the data. Data are presented as mean ± SEM.

### Computational methods

#### Kinetic modeling: vgckimo program package^1^

The dominant paradigm for ion transport over the past 60 years has been based on the seminal experiments of Hodgkin and Huxley (Hodgkin and Huxley, 1952; Hodgkin et al., 1952). However, a much more detailed picture of the mechanisms underlying membrane excitation can be described in terms of Markov models (M-models) (Rudy and Silva, 2006; Moreno et al., 2011), where the conducting and non-conducting states are interconnected by rate constants dependent on the membrane potential. The essence of M-models is that, for any single step in the gating mechanism, the transition probability (i.e., the microscopic equivalent of the rate constant) is time independent. In an M-model of ion channels, transition rates define the interstate dynamics. These rates may depend on environmental variables such us membrane potential or ligand concentration.

The state probabilities in the model are calculated by solving the following differential equation (Equation. 2):

(2) $$\begin{array}{r} \frac{d\vec{p}}{dt}=Q\vec{p} \end{array}$$

where $\vec{p}$ is the vector of state probabilities and *Q* is the system matrix of the transition rate constants. Each transition rate constant is assumed to have the following expression:

(3) $$\begin{array}{r} k_{i}=\alpha_{i}e^{\beta_{i}V} \end{array}$$

where $\alpha_{i}=\frac{k_{B}T}{h}•e^{\frac{\Delta S_{i}}{R}-\frac{{\Delta H}_{i}}{RT}}$ (*ms*^−1^), $\beta_{i}=\frac{z_{i}F}{RT}$ (*mV*^−1^); *V* is the external electric potential in *mV*; z_i_ is the effective valence of moving charges; T(K) is the temperature; ΔH_i_ (J/M) the change in enthalpy; ΔS_i_(J/M/K) the change in entropy. k_B_ = 1.381 10^−23^ J K^−1^ (Boltzmann constant); h = 6.626 10^−34^ J s^−1^ (Planck constant); *R* = 8.315 Jmol^−1^ K^−1^ (ideal gas constant); *F* = 96785 C M^−1^ (Faraday constant).

Once Equation (2) is solved, the probability of being in the open state (p_O_: conducting state) is found and the current is calculated using the following equations:

(4) $$\begin{array}{r} I_{Kr}=g_{Kr}p_{O}\left(V-E_{k}\right) \end{array}$$

(5) $$\begin{array}{r} g_{Kr}=g_{Kr}^{0}\left(aT+b\right){\left(\frac{{\left[K+\right]}_{O}}{5.4\;mM}\right)}^{\frac{1}{2}} \end{array}$$

Where $g_{Kr}^{0}=$0.024 pA/pF/mV, a = 1/35, and b = −55/7, O is the open probability (see SM and (Fink et al., 2008) for more details).

Through the Global-Fitting procedure described in Balser et al. (1990) and implemented in VGC-KiMo^1^, the rate constants of a given M-model can be estimated from macroscopic ion channel currents in voltage-clamped membranes. The use of comprehensive and extensive data sets of experimental information from a broad range of ion current responses to multiple voltage stimulations conditions (voltage protocols, membrane potentials, temperature, etc.), shrinks the universe of possible solutions to the model system mechanism ensuring the robustness of the parameter set. Although several methods exist for analyzing voltage dependent currents (Wang et al., 1997; Mazhari et al., 2001; Fink et al., 2008; Bett et al., 2011; Moreno et al., 2011; Ben-Shalom et al., 2012) most of them are published only as a set of equations without the simulation tools. Others, from the neurophysiology field, are designed to use the full current traces, data that are neither commonly available nor easy to extract from published literature (Gurkiewicz and Korngreen, 2007; Ben-Shalom et al., 2012).

In the current form, VGC-KiMo^1^ source code includes two Markov formulations for the Kv11.1, best known as the hERG K^+^ channel. Any other channel can be added to the source code in addition to the current one, as well as different Markov models and other voltage protocols. The experimental data chosen for the model validation was not used for the development of the model's parameters and belongs to a different cell line than the one used to originally derive the parameters for the M-model (HEK cells), see SM (Section 2: Validation). The performance of the original parameters is fairly good but corrections were needed to reproduce the data from CHO cell line, suggesting that the published set of parameters is robust and reliable. A preliminary version of VGC-KiMo has also been used recently to simulate WT hERG and a variant using experimental data from HEK cell line (Guo et al., 2015; Perissinotti et al., 2015).

#### Cell simulations

An I_Kr_ Markov model (Romero et al., 2015) was incorporated into the O'Hara-Rudy human ventricular action potential model(O'hara et al., 2011) and its maximum conductance (gKr = 0.0422) was scaled to elicit a close value of the peak Ikr as the original O'Hara model at 1 Hz. Physiological action potential simulations were subsequently performed at 37°C. b-Isoform and a-Isoform transition rate constants together with the corresponding temperature correction are shown in Table S12.

Simulated action potentials (APs) were recorded in endocardial cells at the 1000th paced beat (BCL = 1000 ms). The numerical method used for updating the voltage was forward Euler. All the Simulations were encoded in C/C++ and run on Mac Pro 3.06 GHz 12-Core computers. The time step was set to 0.00005 ms during AP upstroke, otherwise the time step was 0.005 ms. Numerical results were visualized using MATLAB R2014a by The Math Works, Inc.

##### Transmural fiber simulations

We simulated a transmural fiber composed of 165 ventricular cells (Δx = Δy = 100 μm) connected by resistances to simulate gap junctions (Faber and Rudy, 2000). The fiber contains an endocardial (cells 1 to 60), M-cell (cells 61 to 105), and epicardial (cells 106 to 165) regions, as described by O'Hara et al. The fiber was paced at BCL = 1,000 ms for 2,000 beats. The stimulus was applied to the first cell. Current flow is described by the following equation:

(6) $$\begin{array}{r} \frac{\partial V\left(x,t\right)}{\partial t}=D\frac{\partial^{2}V\left(x,t\right)}{\partial x^{2}}-\frac{I_{ion}\text{\_}I_{stim}}{C_{m}} \end{array}$$

Where *V* is the membrane potential, *t* is time, *D* is the tissue diffusion coefficient [0.00092 cm^2^/ms, calculated from Shaw and Rudy (Shaw and Rudy, 1997)], *I*_*ion*_ is the sum of transmembrane ionic currents, *I*_*stim*_ is the stimulus current (300 μA/cm^2^ for 0.5 ms), and *C*_*m*_ is the membrane capacitance (1 μF/cm^2^).

##### Ecg computation

Extracellular unipolar potentials (Φ_e_) generated by the fiber in an extensive medium of conductivity σ_e_, were computed from the transmembrane potential *V*_*m*_ using the integral expression as in Gima and Rudy (Gima and Rudy, 2002):

(7) $$\begin{array}{r} \Phi_{e}\left(x^{\prime },y^{\prime },z^{\prime }\right)=\frac{a^{2}\sigma_{i}}{4\sigma_{e}}\int \left(-\nabla V_{m}\right)•\left[\nabla \frac{1}{r}\right]dx \end{array}$$

(8) $$\begin{array}{r} r={\left[{\left(x-x^{\prime }\right)}^{2}+{\left(y-y^{\prime }\right)}^{2}+{\left(z-z^{\prime }\right)}^{2}\right]}^{1/2} \end{array}$$

where ∇*V* is the spatial gradient of *V*_*m*_, *a* is the radius of the fiber, σ_*i*_ is the intracellular conductivity, σ_*e*_ is the extracellular conductivity, and *r* is the distance from a source point (x, y, z) to a field point (x', y', z'). Φ_*e*_ was computed at an “electrode” site 2.0 cm away from the distal end along the fiber axis.

### Structural modeling

Recently published full hERG (hERG_T_) open channel solved by Cryo-EM at 3.8 angstrom resolution (PDB ID 5VA2) was used for the structural analysis. The construct used for structural studies has functional properties very similar to WT but is lacking residues between 141 and 350, that correspond to the structure between PAS and S1; and 871-1005 (C-terminal). The structure was cut right after CNBD ends and missing residues at the outer pore mouth were added and modeled as extracellular loops that were minimized using NAMD2.10 (Phillips et al., 2005). The 3D structure of the closed-state hERG channel used in this study is based on the homology modeling to EAG1 Cryo-EM structure (PDB ID 5K7L) determined at 3.78 angstrom resolution (Yang et al., 2017). This structure represents the closed pore while the voltage sensing domain (VSD) displays an open conformation (Whicher and Mackinnon, 2016). The SWISS-MODEL homology modeling program (Arnold et al., 2006) was used for the development of the hERG closed model from the available EAG1 channel structure as described previously (Yang et al., 2017). Sequence alignment was performed using the CLUSTALW algorithm (Larkin et al., 2007; Goujon et al., 2010). Protein models were generated from the alignment in a stepwise manner. The generated model was later minimized using NAMD2.10 (Phillips et al., 2005).

## Results and discussions

### Experimental measurements

The whole-cell patch clamp configuration at room temperature was used to study the voltage-dependent activation by applying a standard step protocol described in the methodology. The normalized tail currents measured at −100 mV were plotted against the membrane potential of the previous step and fitted to a Boltzmann function. The V_1/2_ of activation is shifted around 10 mV in the negative direction for hERG1b compared to hERG1a (Figure 3, Table 1). The mean current levels measured at the end of the 1-s depolarizing pulse to +40 mV were used to construct the current-voltage (I-V) relationship (Figure 3C). Similar to what is observed for hERG1a, the b-isoform shows a strong inward rectification, resulting in the characteristic bell-shaped curve. Kinetics of activation was studied by applying an envelope of tails protocol, as described in the methodology section. The b-isoform shows a similar sigmoid shape of activation, but a much faster rate compared to the a-isoform (Figure 4).

**Table 1:** Experimental data for HEK cells at room temperature.

	**hERG1a**	**hERG1b**
**ACTIVATION PARAMETERS**		
V_1/2_ (mV);	−10.7 ± 1.5 (*n* = 10)	−20.9 ± 2.4 (*n* = 10)
slope (mV)	8.1 ± 0.3	8.8 ± 0.8
**RECOVERY FROM INACTIVATION**, τ **(ms)**		
−100 mV	3.6 ± 0.2 (*n* = 7)	1.1 ± 0.1 (*n* = 6)
−50 mV	11.0 ± 0.8 (*n* = 4)	2.1 ± 0.5 (*n* = 3)

*Each value is an average of n experiments. Equation (1) was used to obtain V_1/2_ and k, data are presented as mean ± SEM*.

Deactivation kinetics was characterized by recording tail currents at potentials ranging from −40 to −120 mV after an activating step to +40 mV (see methods). Current traces for selected voltages are shown in Figure 5. The deactivating currents were best fitted to a double exponential function and time constants corresponding to the fast and slow deactivation processes are shown in Table S13. Both deactivating components are significantly reduced for hERG1b. The observed reduction depends on the voltage, at −60 mV, the slow and fast components are around 10 to 14 times faster for hERG1b while the difference is around 4 to 7 times for −40 and −100 mV, respectively. As it was found before for CHO cells by Larsen et al. (2008), the relative contribution of the fast component of deactivation depends on the voltage and is much more pronounced for hERG1b compared to hERG1a, in fact at −120 mV there is no slow component according to the experimental fit. Regarding the inactivation process, there was not significant difference and was not further investigated here (Larsen et al., 2008) (data not shown). Time constants for recovery from inactivation for a-isoform and b-isoform display a significant difference and are collected in Table 1.

### Markov kinetic models to describe hERG a- and b-isoforms

The most pronounced difference evident from the experimental raw current traces is the markedly faster deactivation rate and the faster activation rate of hERG1b compared to hERG1a. This is in agreement to what was also found for CHO cells (Larsen et al., 2008). The experimental measurements in the HEK cell line focus in these events and the M-models were fitted to the data presented in the above section. All transition rates were defined using Equation (3). The previously derived values for α_i_ and β_i_ were used for initial guess (see Table S1 in Supplemental Materials). For M-model 1, the initial guess values were derived using the comprehensive experimental data-set from Berecki et al. at room temperature and 37°C respectively (Berecki et al., 2005). Two correction terms, *a* and *b* were introduced to α and β parameters for quality monitoring during optimization routine (Equation 9). α and β were set to the constant values, while ***a*** and ***b*** parameters were introduced as free variables for optimization routine.

(9) $$\begin{array}{r} k_{i}=a_{i}\alpha_{i}e^{b_{i}\beta_{i}V} \end{array}$$

Correction to individual parameters (**a**_i_, **b**_i_: Equation 5) from Fink et al. and Mazhari et al. has been done to reproduce available data from HEK-cell measurements at room temperature (23). All fitted parameters for the kinetic mechanism considered, can be found in Tables 2, 3 and Tables S4, S5. Note that β was set to zero for transitions *a*_*in*_ and *b*_*in*_ (Figure 2) and thus, these transitions are modeled as voltage independent. Maximum single channel conductance was assumed to be the same for hERG1a and hERG1b, so all differences are attributed to channel kinetics.

**Table 2:** M-model 1 rate constants for transitions within hERG gating for a-isoform and b-isoform.

**Transition**	**Parameter name**		**Rates**		**ratio b/a**
			**b-Isoform**	**a-Isoform**	
C3 → C2	ae	α	4.71 E-2	3.39 E-2	**1.39**
		β	9.36 E-3	1.04 E-2	0.90
C2 → C3	be	α	7.43 E-2	4.82 E-2	**1.54**
		β	−5.05 E-2	−6.91 E-2	0.73
C2 → C1	ain	α	5.01 E-2	2.20 E-2	**2.28**
C1 → C2	bin	α	2.95 E-2	1.36 E-2	**2.17**
I → O	**ai**	α	2.80 E-2	6.63 E-3	**4.23**
		β	−3.06 E-2	−3.89 E-2	0.79
O → I	bi	α	7.41 E-2	7.41 E-2	1.00
		β	1.88 E-2	2.80 E-2	0.67
C1 → O	**aa**	α	1.71 E-2	4.94 E-3	**3.46**
		β	3.05 E-2	4.31 E-2	0.71
O → C1	**bb**	α	1.61 E-3	2.06 E-4	***7.83***
		β	−3.44 E-2	−3.76 E-2	0.91

α (1/ms) indicates voltage independent rate, and β (1/mV) indicates voltage dependent rate as follows: k = α^*^exp(β^*^V) The transitions between O and I states are also dependent on extracellular potassium concentration [K^+^]^0^. That dependence is accounted for in the model by modifying the transition rate for inactivation (O → I, bi) as: k_bi_([K^+^]^0^) = k'_bi_(5.4mM/[K^+^]^0^)^0.4^. Bold values indicate moderate change of the corresponding transitions calculated as a ratio. Transitions that are strongly affected are shown in bold red. Transitions that are affected the most are shown as underlined red bold values. For details of the optimizations, original model parameters and optimization from CHO data see Tables S1–S4.

**Table 3:** M-model 2 rate constants for transitions within hERG gating for a-isoform and b-isoform.

**Transition**	**Parameter name**		**Rates**		**ratio b/a**
			**b-Isoform**	**a-Isoform**	
C3 → C2	ae	α	1.19 E-2	3.77 E-3	**3.17**
		β	2.17 E-2	3.29 E-2	0.66
C2 → C3	be	α	1.82 E-2	2.44 E-2	0.74
		β	−3.79 E-2	−6.77 E-2	0.56
C2 → C1	ain	α	9.29 E-2	3.59 E-2	**2.59**
C1 → C2	bin	α	1.09 E-1	1.65 E-2	**6.62**
I → O	**ai**	α	5.11 E-2	1.74 E-2	**2.93**
		β	2.09 E-2	2.87 E-2	0.73
O → I	bi	α	1.79 E-2	9.26469 E-3	1.94
		β	−2.15 E-2	−2.347 E-2	0.92
C1 → O	**aa**	α	1.55 E-1	8.80 E-2	**1.76**
		β	9.60 E-3	1.02 E-2	0.94
O → C1	**bb**	α	7.82 E-2	5.11 E-3	**15.28**
		β	−3.54 E-2	−4.44 E-2	0.80
C1 → I	bi^*^	α	9.32E-07	1.02E-09	**910.41**
		β	1.621E-05	7.99E-06	2.03

α (1/ms) indicates voltage independent rate, and β (1/mV) indicates voltage dependent rate as follows: k = α^*^exp(β^*^V).

* *Constraint by microscopic reversibility. Bold values indicate moderate change of the corresponding transitions calculated as a ratio. Transitions that are strongly affected are shown in bold red. Transitions that are affected the most are shown as underlined red bold values. For additional details on the optimizations and original model parameters see Tables S1–S4*.

Three and/or four different voltage protocols were included simultaneously in the optimization protocol (see Supplementary Materials for a complete description of the voltage protocols). Different optimization routines were performed and the correction factors were defined for each of the parameters. All parameters required corrections to the initial guesses. The best fits are shown in Tables 2, 3 for two isoforms and each model, respectively. Further improvement of the optimizations was achieved by using the random initial guess generator around the already fitted values and assigning different weight to the partial cost function. In all the cases, the stability analysis showed that all values were well-converged. The simulated voltage protocols and computed currents are in reasonable agreement with the measured ones, meaning that voltage dependence and curve shapes are qualitatively well-reproduced. Overall, both gating mechanisms from the literature were able to reproduce the gating kinetics for both isoforms. However, the optimized parameters showed some interesting differences and limitations in quantitatively reproducing the time course of the deactivation kinetics at different voltages.

#### Performance of M-model 1

**Figure 6:**
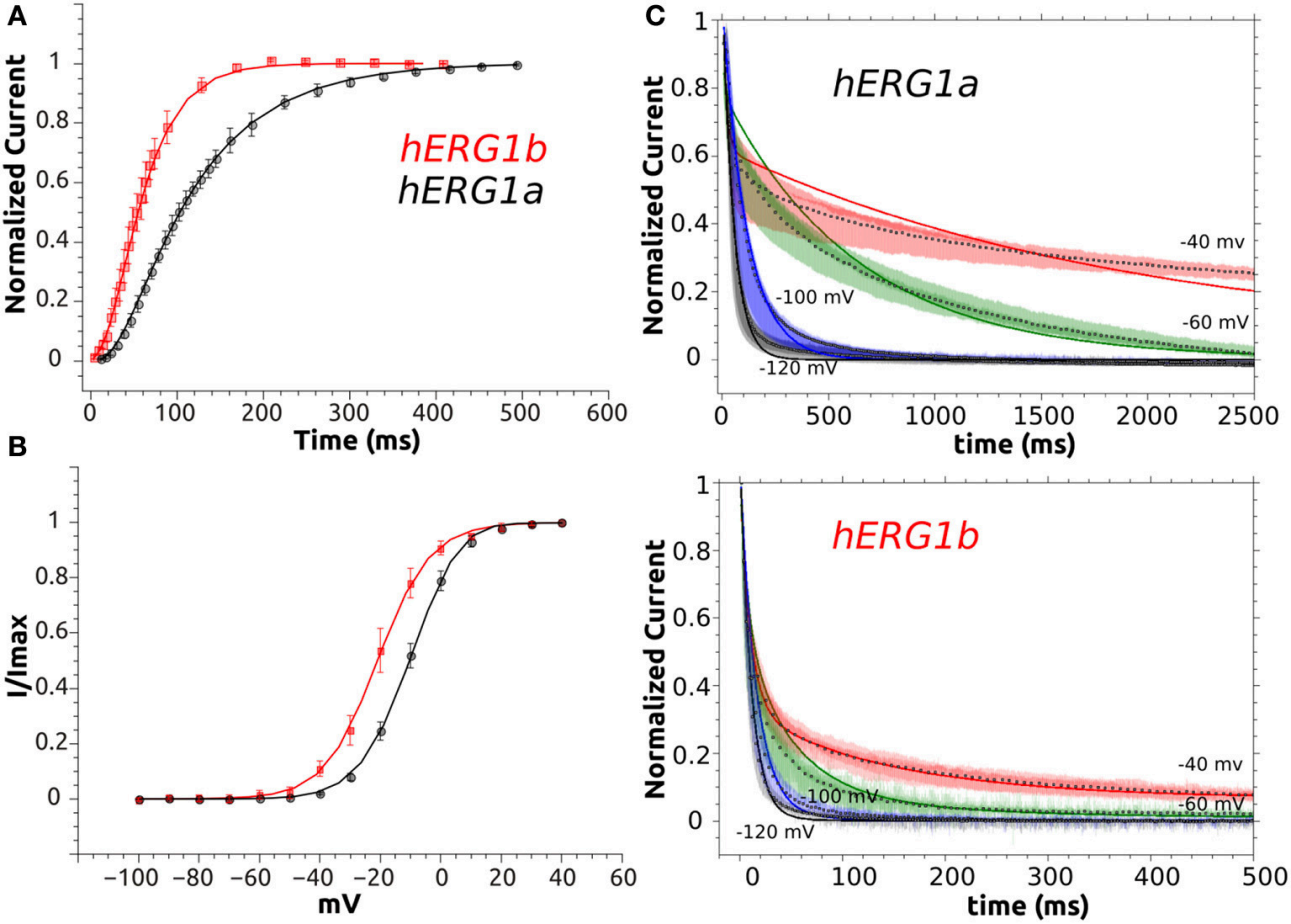
M-model 1 optimization to experimental data. **(A)** Activation curve of a-isoform and b-isoform. **(B)** Steady State Activation curve for both isoforms. **(C)** Simulated deactivation curves at −120, −100, −60, and −40 mV. Simulated data is shown as solid lines and symbols are used for experimental time course data (average, *n* = 10). Superimposed raw time course data (*n* = 10) is shown in the background for deactivation at each voltage.

The M-model 1 implies linear connectivity between different gating states. The channel has to go through the open state to fully inactivate. The experimental basis for this scheme was extensively discussed in the literature (Bett et al., 2011). Assuming that both isoforms follow same kinetic scheme (Sale et al., 2008), the key differences between the a-isoform and b-isoform kinetics lie essentially in the highlighted steps shown in Table 2. According to the results collected in Table 2, the best fit within M-model 1 indicates that the main difference between two hERG isoforms is in the late deactivation step. The late deactivation is about eight times faster for the b-isoform. These parameters also show an increase in the activation rate steps, together with an increase in the recovery from inactivation rate in agreement with the experimental data (Table 1). Simulated data from CHO cell line (SM) also display a good agreement with the optimal fit (Tables S3, S4, S9). Figure 6 shows the simulated data together with the experiments. An excellent agreement is obtained for the activation and Steady State Activation curves for both isoforms (**Figure 9**). However, the main challenges are in the modeling of the deactivation kinetics for different voltage protocols (−40, −60, −100, −120 mV). For b-isoform in particular, the quality of the optimization is limited, although it qualitatively reproduces the behavior at the different voltages.

#### Performance of M-model 2

**Figure 7:**
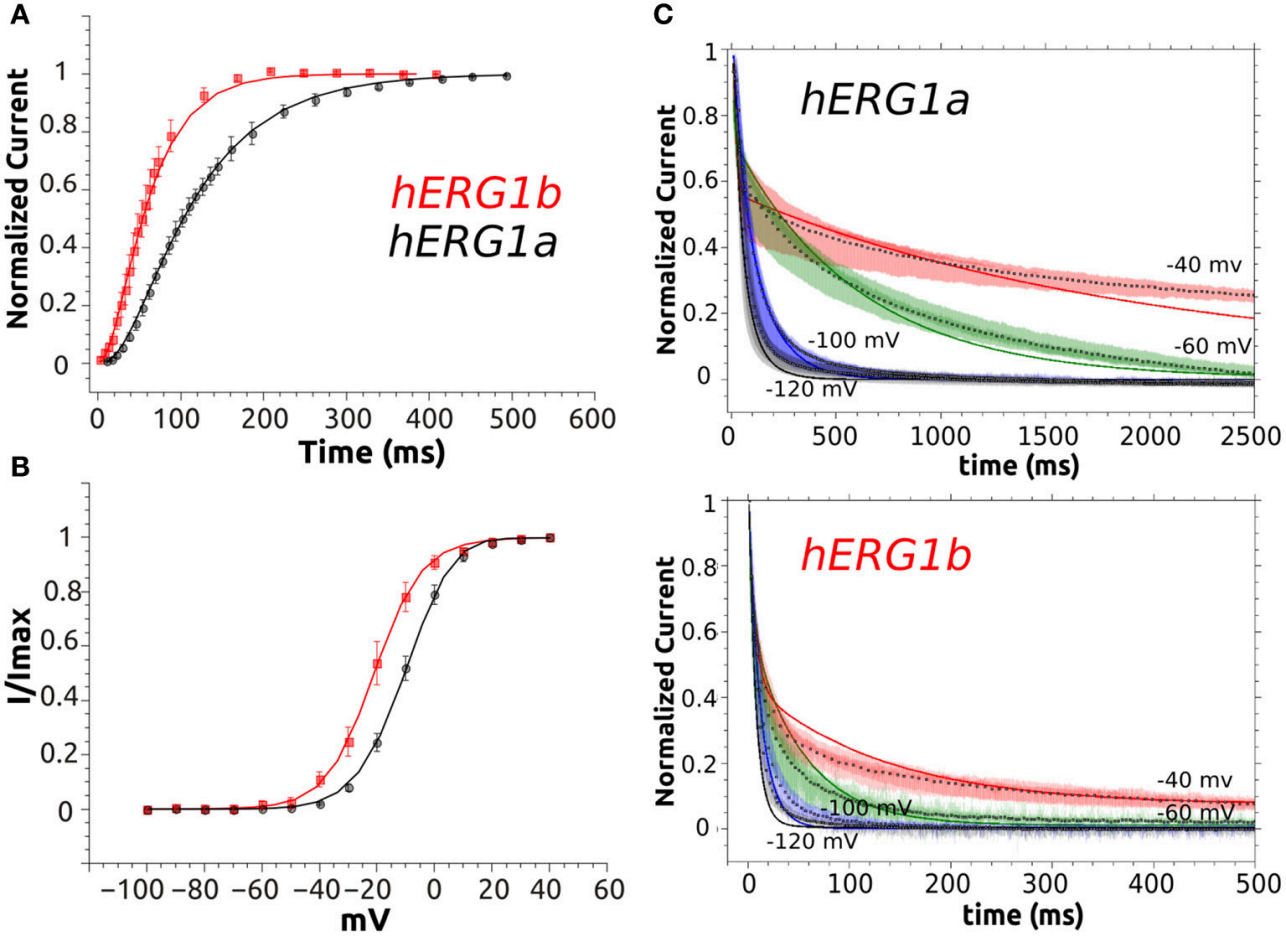
M-model 2 fit to experimental data. **(A)** Activation curve of a-isoform and b-isoform. **(B)** Steady State Activation curve for both isoforms. **(C)** Simulated deactivation curves at −120, −100, −60, and −40 mV. Simulated data is shown as solid lines and symbols are used for experimental time course data (average, *n* = 10). Superimposed raw time course data (*n* = 10) is shown in the background for deactivation at each voltage.

This model, in contrast to the M-model 1, is not linear, but instead, includes a direct transition to the inactivated state from the closed state immediately preceding the open state. In one of the previous formulations (Mazhari et al., 2001), this transition is negligible compared to the transition to the open state and so, numerically, this model is almost linear. In our work, M-model 2 was tested assuming a range of different values for this transition and in all the cases a very small rate was obtained for both isoforms. Although the obtained value was small and almost negligible, it is almost 1000 times faster for the b-isoform. The M-model 2 shows a similar performance compared to the M-model 1 although the fit quality is consistently lower than that of M-model 1. The differences between a-isoform and b-isoform are distributed over the activation steps but mainly in the late deactivation and the new extra step considered in this scheme (Table 3). Simulated current traces elicited by the voltage protocols and the activation curves are well-reproduced by this model (Figure 7). Similar to what was observed for M-model 1, the quality of the optimization is poor, although it qualitatively reproduces the behavior at the different voltages.

**Figure 8:**
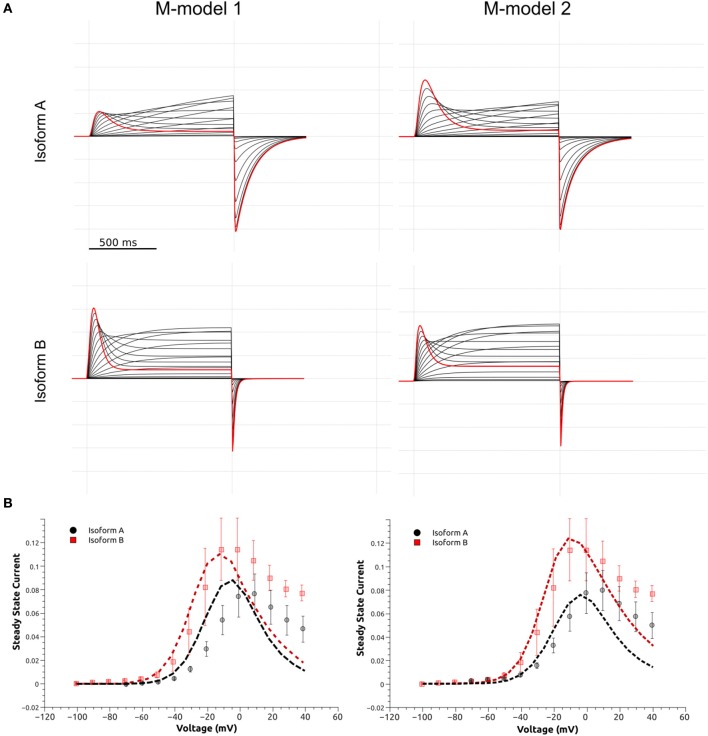
Simulated currents M-model 1 and M-model 2. **(A)** Simulated currents elicited by the SSA voltage protocol (Figure 3) for both M-models and Isoforms. **(B)** Simulated I-V steady state current (x10^4^ pA) relationship for M-model 1 and 2. a-Isoform is shown as black dashed line and b-isoform as red dashed line. Experimental data is shown in the background as symbols with the corresponding error bar (Figure 3).

Taking together the results from M-model 1 and 2, we conclude that both models capture the main kinetic difference between the isoforms. The pivotal feature of isoform kinetics is a considerable increase in the late deactivation step. Both models point to a moderate increase in the activation and recovery from inactivation steps, the changes and their magnitudes depend on the model. The current traces simulated by each model and elicited by the SSA voltage protocol are shown in Figure 8. It can be seen that they qualitatively reproduce the experimental behavior shown in Figure 3. The b-isoform displays larger currents, an increased activation rate, faster recovery from inactivation and clearly shows a much faster deactivation rate under the repolarizing pulse. The simulated I-V relationships show the typical curve shape and qualitatively reproduce the experimental differences characteristic of each isoform, although they show significant deviations for voltages above 10 mV. At these voltages background currents are relatively high, while hERG current is relatively small. The combination of these two factors presents a natural challenge and led to the discussed discrepancy between simulated and experimental data.

The best optimization for M-model 1 which also shows fair agreement with the fit to CHO data (Table S9), suggests that although the main difference is in the late deactivation step, being eight times faster for the b-isoform when compared to a-isoform, early and late activation are also increased by a factor of ~2 and 4, respectively. According to this fit, the voltage independent rates are increased in forward and backward directions by a factor of 2 and the recovery from inactivation rate by a factor of 4. M-model 2 suggests similar changes and fair agreement with CHO data (Table S10); late deactivation is 15 times faster for the b- compared to the a-isoform, but also early and late activation, being 3 and 2 times faster respectively. It also shows a three-fold increase for the recovery from inactivation and an increase for both voltage insensitive rates, being 2.5 times faster in the deactivating direction.

#### Limitations of the kinetic modeling

It is important to mention that the time course of deactivation was not well-fitted by the M-models used in this work for both HEK and CHO cell lines. We observed that the quality of the fit is different for different voltages. To improve modeling of the deactivation kinetics, a number of different conditions were tested for both models, i.e., randomization of initial values, constraints, boundaries, etc., but no further improvement was achieved. The reason could be related to the amount of data used in the fitting procedure, experimental limitations in the data acquisition (temperature, cell-line variability, resolution of electrophysiological recordings), or indicate that the M-models should be revisited. It is important to mention that some inconsistencies between the deactivation experimental data and these models were previously discussed by other authors (Fink et al., 2008). Another example can be found in a recently developed Markov model that reproduces biophysical experimental data at room temperature with CHO cell line includes two closed (C1, C2), one open (O) and corresponding inactivated states (IC1, IC2, IO) (Di Veroli et al., 2013). This model also faces difficulties in fitting deactivation at different voltages. Interestingly, the 6-states model with closed loops can be reduced to a 4-states cyclic model (C1, O, IC, IO) at 37°C, as different closed states could not be resolved. However, unless explicitly introducing temperature-dependent parameters, all of the available models cannot account for temperature-dependent hERG channel activity changes. A modification of the Di Veroli model was done recently by Li et al. (Li et al., 2016), and can recapitulate macroscopic hERG channel gating behavior for a temperature range from 20 to 37°C. Providing the better performance for the temperature range, different states and connectivity, it would be interesting to test the performance of this new model in reproducing the experimental data for both isoforms. It is important to emphasize that having a complete and more reliable M-model is of key importance for modeling and predicting differential and temperature dependent effects of drugs on the delayed rectifier potassium “I_kr_” current.

### Structural underpinnings of isoform function

**Figure 9:**
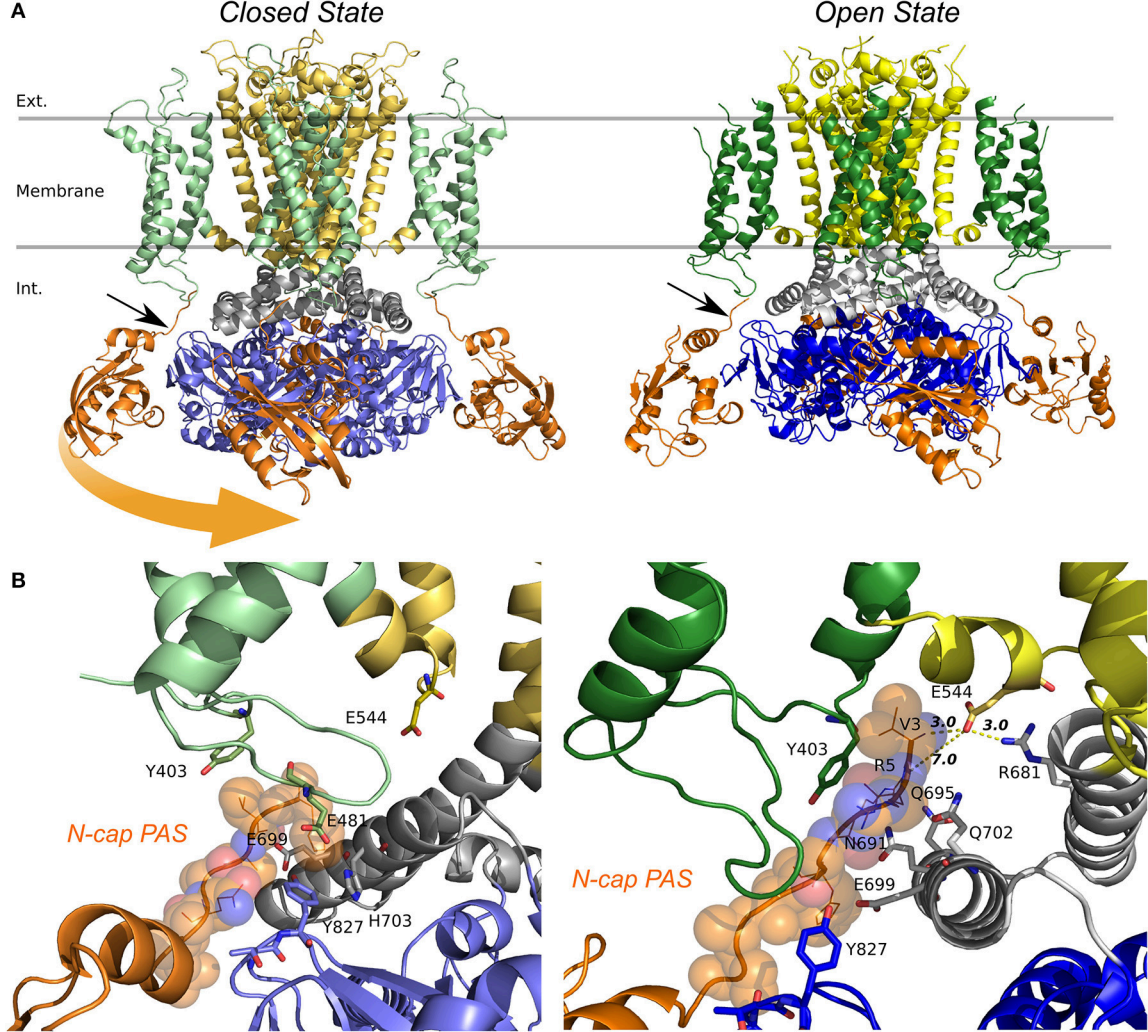
Models of the quaternary structure of homomeric hERG tetramers. Side view **(A)** of closed state model in the left panel and open state hERG Cryo-EM structure, right panel. In both structures N-cap PAS can be seen interacting with VSD (black arrow). When the TM domain is aligned, cytoplasmic domains are slightly rotated respects each other. Orange arrow show the rotation direction when transitioning from closed to open states. **(B)** N-cap PAS interaction with VS (S1 & potentially S4-S5), CNBD, and C-linker for closed in the left panel and open states, right panel.

The kinetic modeling discussed above isolates principal differences in gating kinetics of hERG a- and hERG b-isoforms. The recent Cryo-EM structures allowed the structural modeling for open- and closed states of hERG1 channel enabling molecular-level description of the determinants of this apparent isoform-specific differences. Homology models (Wacker et al., 2017) and chimera constructs were very useful in the past (Dhillon et al., 2014) for understanding structure-function relationships in K+ channels. However, most of the models were focusing on the trans-membrane section of hERG1 channel only (Wacker et al., 2017). The recently-solved hERG structure shows an open pore, while the EAG1 channel solved by Cryo-EM is captured with the pore closed due to the presence of Ca2+ and calmodulin, which lock the pore closed while the VSD is supposed to be in its depolarized state (Wang and Mackinnon, 2017). The hERG closed model presented here was built using EAG1 structure as a representative template for hERG‘s closed pore. Given the fact that conformational differences between these two states of VSD are relatively small compared to structures and models of open- and closed states found in K^+^ channels from Shaker family (Li et al., 2014); then the question is, if this is a good representation of hERG closed state, what kind of VSD movement could result in that same conformational change in the pore?. As Wang et al. (Wang and Mackinnon, 2017) pointed out, there are key structural differences in the arrangement of the VSD (non-domain swapped) in hERG and EAG1. It seems that an S4 inward movement toward the cytoplasm and centric displacement toward the pore axis driven by the membrane electric field could produce a similar pore closure. In that scenario, there is almost not translation of S4 across the membrane, S5 maintains an extensive antiparallel contact with S6 and the VSD would transmit force through the S5-S6 interface as the movement of S4 would compress the S5 helices and close the S6 gate. This proposed mechanism is different than the lever mechanism proposed for Shaker-like Kv channels and the cytoplasmic domains may play a crucial role in it. It is important to mention that functional measurements also point to substantial differences in the total gating charge, being much less for hERG, which implies that the VSD conformational changes are smaller in hERG channel (Zhang et al., 2004; Li et al., 2014). These rapid developments in hERG structural biology emphasized important roles of PAS and CNBD domains in gating kinetics. As it was shown previously for Kv1.2-Kv2.1 (Morais-Cabral and Robertson, 2015), PAS-CNBD complex published for mEAG1 (Haitin et al., 2013), and all the recent structures; the PAS (Figure 9, in orange) domain is far away from the VSD. In stark contrast, hERG structures show (Whicher and Mackinnon, 2016; Wang and Mackinnon, 2017), that the N-terminus of the PAS domain (absent in b-isoform) is directed toward the VSD and S4-S5 linker (Figure 9B) and most likely interacts with the gating machinery. NMR studies previously suggested that the N-terminal cap shows a high degree of structural variability and is long enough to reach the voltage sensor, the S4-S5 linker or the C-linker (Muskett et al., 2011; Ng et al., 2011, 2014) (Figure 9B). As it was mentioned before, the new structures present new topology of VSD-pore domain packing, which is different from the domain-swapped architecture and might suggest a new paradigm for voltage dependent gating. It was recently proposed for EAG1, a mechanism in which the VSD interacts with the cytoplasmic domains to gate the channel. Combined with the data from isoform kinetic modeling described above, models of hERG in open and closed state may provide better understanding of stabilizing interactions present or missing in a particular isoform.

**Figure 10:**
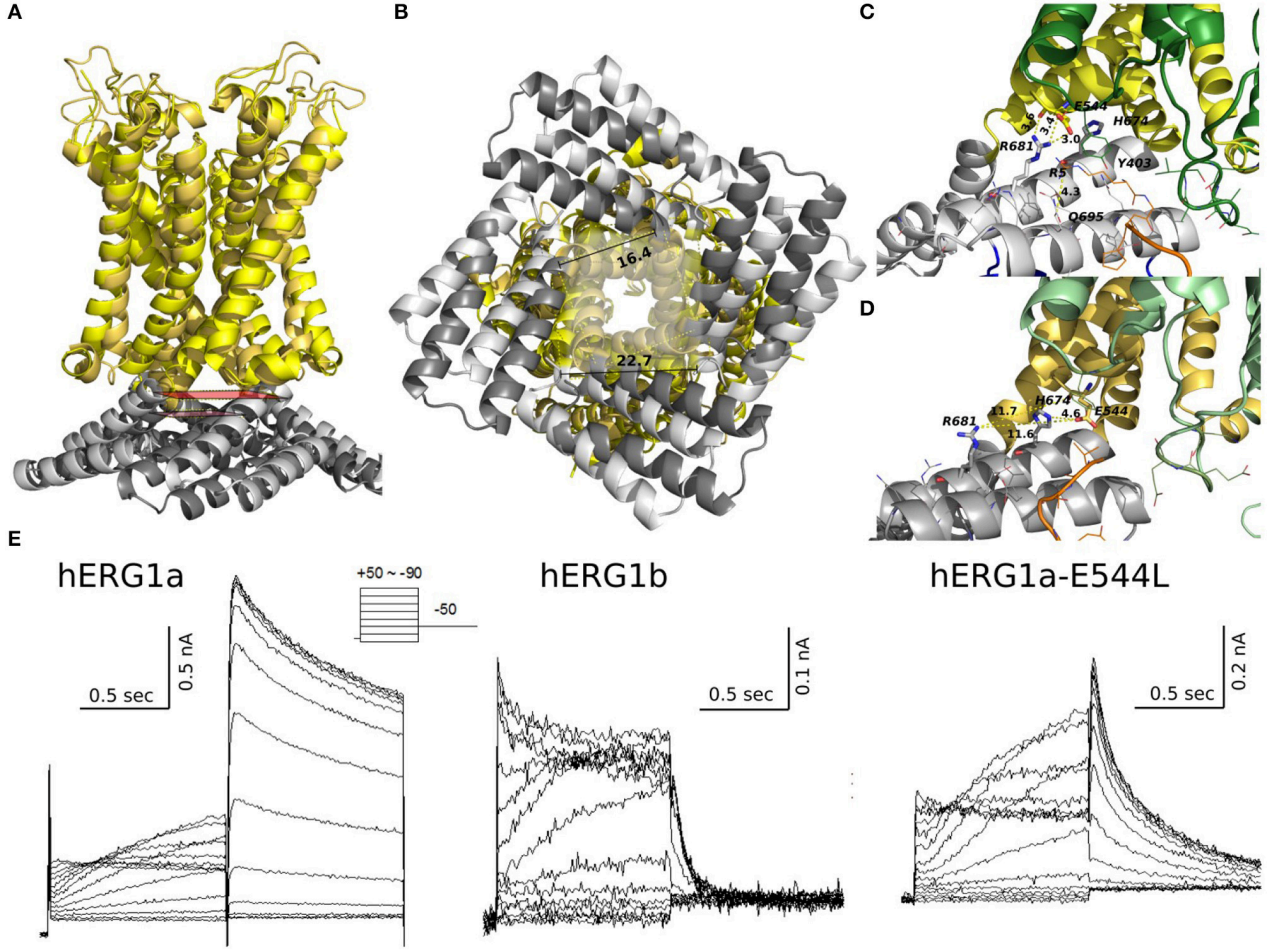
Structural alignment of closed (light colors) and open (dark colors) models of hERG. TM and C-linker side view shows that the C-linker/TM are closer to each other in the open state compared to the closed **(A)**. Distances between residues Gly669 show that a closer C-linker is a consequence of a more open pore in the open state **(B)**. Key salt bridge interactions between Glu544 and Arg681 can only be found in the open state **(C)** but not in the closed state **(D)**. **(E)** Current traces from experiments previously published by Durdagi et al. for hERG1a, hERG1b, and hERG1a-E544L elicited by the voltage protocol at the top. E544L mutant displays a faster deactivation rates than WT but not as much as hERG1b.

The arrangement of cytoplasmic domains in the open state and closed state models are shown in Figure 9. The PAS domain is interacting with CNBD in a similar way it was found previously for homologous channels (Lee and Mackinnon, 2017; Li et al., 2017; Wang and Mackinnon, 2017). Similar to the previously solved structures for CNBD domains (Ng et al., 2011; Adaixo et al., 2013; Brelidze et al., 2013; Haitin et al., 2013), a portion of the hERG sequence occupies the cyclic nucleotide binding site, which prevents the cyclic nucleotide binding. In addition to that, the N-terminus of PAS Domain (N-cap), which influences the rate of voltage dependent channel opening and closing, is directed toward the VSD (Wang and Mackinnon, 2017) (Figures 9B, 10B). When the channel is in its open state, the C-linker region is packed against the transmembrane domain (Figure 10A) interacting with the S4-S5 linker and VSD. A novel interaction pinpointed by the structural analysis is the salt-bridge formed between Glu544 and Arg681 (Figures 10C,D). This salt-bridge is missing in the closed state model of the channel as the C-linker is slightly rotated with respect to the S4-S5 linker. Hence, we hypothesize that it might be one of the open-state stabilizing interactions that it is affected in the absence or the PAS domain (b-isoform), note that N-cap (Val 3) is close to E544 and might indirectly affect the E544-R681 interaction. Unfortunately, solved structures are missing significant part of the PAS domain sequence and further refinement of hERG1 PAS domain is essential future goal for structural modeling. Nevertheless, the previously studied E544L mutant (Durdagi et al., 2012) shows an increase in the deactivation rate. Even though is not as much as for the b-isoform (Figure 10E), it highlights a potential key role of this residue. When transitioning from closed to open state, the transmembrane and cytoplasmic domains slightly rotate with respect to each other (Figures 9, 10B), we suggest that the interplay between PAS, VSD: S4-S5, and C-linker during such rotation might be of key importance in modulation the gating.

**Figure 11:**
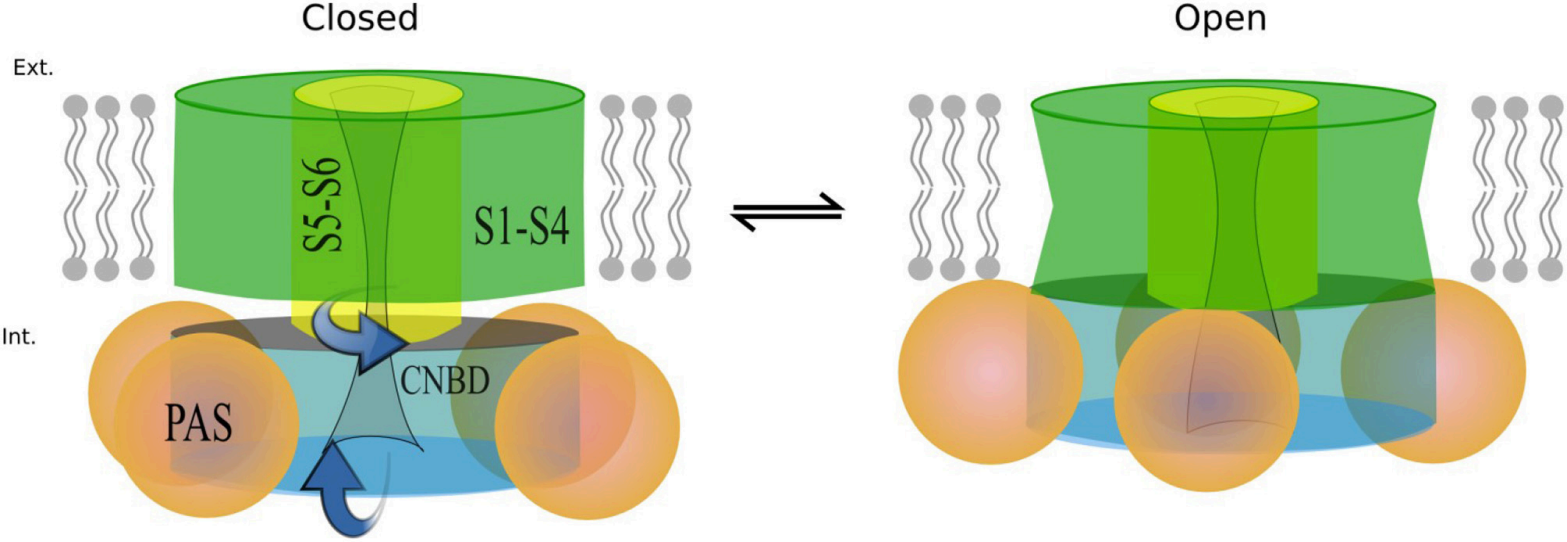
Proposed mechanism based on the structures modeled. When the channel opens the cytoplasmic domains rotate toward the membrane as the S6 end twists in the same direction.

While more work is still required to decipher gating kinetics of hERG1, the structural models already show enhanced interactions between the cytoplasmic and the transmembrane (TM) domain for the open state of the channel. Analysis of structural differences between open and closed states suggests that a slight rotational movement changing packing of the cytoplasmic domains against the TM part of the channel is required as part of activation/deactivation process. This is in line with the finding that the conformational change that VSD is undergoing during gating cycle might be small compared to other potassium channels. This would allow the CNBD to close the channel independent of the VSD conformation (as it was observed for EAG1) and provide an added level of regulation through the interaction of intracellular domains with the voltage dependent gating machinery (Whicher and Mackinnon, 2016). These structural insights, although preliminary, lead us to the hypothetic gating mechanism summarized in Figure 11. The similar mechanism of gating modulated by soluble domains has been proposed for MolK1, a prokaryotic potassium channel lacking the C-linker and PAS domain (Kowal et al., 2014).

These structural models also raise another point. In other channels, PAS and CNBD domains serve a regulatory function in which the binding of small molecules or signaling proteins is transduced into conformational changes. It is not known whether or not this could be happening for hERG. These new models align to what was suggested previously (Morais-Cabral and Robertson, 2015), and that points to the possibility that the C-linker-CNBD-PAS serves as an anchor to correctly position the N-pas terminal cap during the gating process. Can we explain observed differences in the deactivation kinetics between hERG a- and b- isoforms observed with kinetic modeling? Any of the functional alterations due to mutations or truncations in the N-terminal cap or the entire PAS Domain (b-isoform) would ultimately lead to a loss of N-terminal cap position and severely-altered gating kinetics. The lack of stabilizing interactions between soluble and trans-membrane domains is expected to impact the opening probability and stability of the open state. It may explain observed rapid transitions between open and closed states present in the b-isoform.

### Cardiac cell models: functional implications of different isoform ratios in the heart

**Figure 12:**
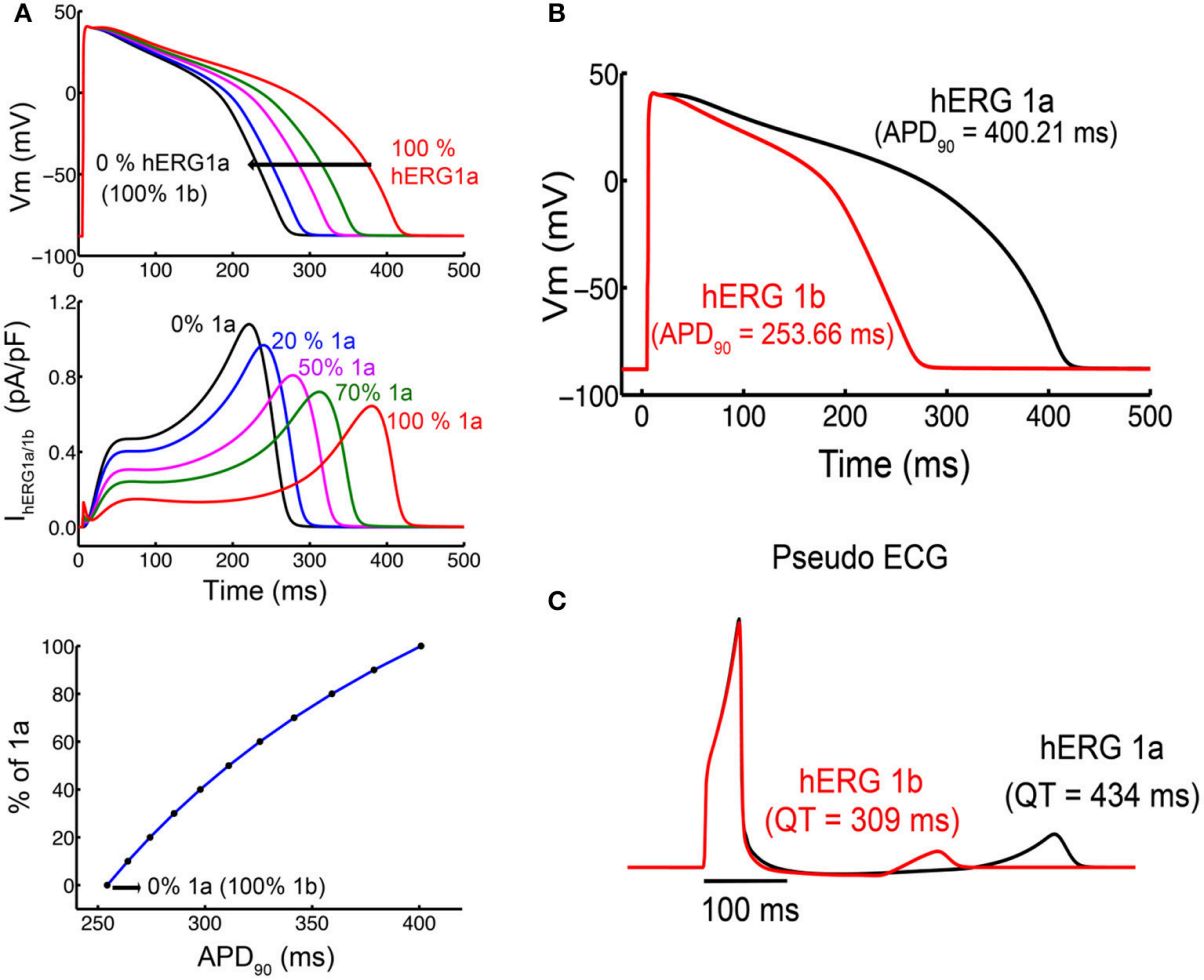
Simulated action potential duration (APD) in cells and virtual pseudo-ECGs in a transmural 1-D tissue model. **(A)** Single cells simulations show concentration response for different ratios of a- and b-isoforms. **(B)** APD for the 1,000th paced beat at 1 Hz in single endocardial cells. hERG 1a shows a longer APD compared to hERG 1b. **(C)** The Pseudo ECGs of hERG 1b (red) indicates shorter QT intervals than hERG 1a.

The improved kinetic models allowed us to directly address physiological questions like whether or not hERG isoform composition in ventricular myocytes has a potential to alter QT duration and, hence, to pre-dispose a patient for drug-induced QT prolongation. To specifically address the functional implication of having homomeric hERG1a or hERG1b in the heart we conducted simulations including our M-model 1 parameters in the cardiac cell (O'Hara-Rudy human cardiac ventricular myocyte Model) (O'hara et al., 2011; Romero et al., 2015). The final parameters from the fittings were then used as input values in the cardiac cell and tissue model (O'hara et al., 2011) in order to simulate the shape of the action potential and ECG signal for both isoforms (Figure 12). The M-model 1 and its parameters from the optimization were introduced in the cardiac cell model in order to test the way in which they affect the shape and duration of action potential. As it was found previously (Larsen and Olesen, 2010), the results of the ventricular cardiomyocyte simulations showed that kinetic changes in I_kr_ corresponding to homomeric hERG1b resulted in much shorter action potential duration (APD). Figure 12B shows the action potential (Dhillon et al.) shape and duration considering the extreme situation of I_kr_ corresponding only to hERG1 a- or b- isoforms. Intermediate cases, where weighed contributions from both isoforms were considered, are shown in Figure 12A. I_kr_ currents are also shown in Figure 12A and, as it can be seen, the currents became larger and peak earlier when transitioning from pure a- to b- isoforms.

Mechanistic inspection of the changes in the channel state occupancy revealed several differences. Figures S9, S11 show the proportion of channels in the different states. The most prominent difference lies in the occupancy of open and inactivated states when comparing a-isoform to b-isoform. The AP shortening is mainly due to an increase in the open state occupancy and a reduction in the inactivated states occupancy for hERG1b compared to hERG1a. Finally, the M-model 1 was also introduced in the tissue model (cable) and the ECG signal was simulated for both homomers (Figure 12C). As expected, virtual hERG1b expression resulted in a reduction in the QT interval on the computed pseudo-ECG.

Experimental investigations have revealed many sources of heterogeneity and associated regulation in the heart. Distinct regions with associated cell types that are distinguishable by morphology and action potential duration have been documented. The different cell types have been shown to arise from heterogeneities in ion channel expression, which have been modeled and used in cell-type specific predictive simulations (Viswanathan et al., 1999) (Shimizu and Antzelevitch, 1999). In the left ventricle of the heart, cellular heterogeneity from the endocardium to mid-myocardium to endocardium exists and arises from heterogeneity in potassium currents (Shimizu and Antzelevitch, 1999) (Liu and Antzelevitch, 1995). The differences in stoichiometry between hERG1a and hERG1b likely constitute a novel source of cardiac heterogeneity that may vary in terms of distribution and be subject to regulation by as yet unknown mechanisms.

The kinetic modeling in section Markov Kinetic Models to Describe hERG a- and b-Isoforms shows a profound difference in hERG1a and hERG1b deactivation rates, where the quantitatively fitted parameters to the data suggested that the hERG1b late deactivation rate is between 8 and 15 times faster than hERG1a. One of the most interesting and counterintuitive findings in the results shown above is that this difference did not result in effects on the action potential duration that would expected from the observed changes alone. The dominant presence of the hERG1 b-isoform results in faster deactivation, which by itself would result in fewer channels in the open state as the channels close more quickly. The anticipated effect on the action potential duration would be less repolarizing current and consequently, shorter APD. In fact, the opposite was observed both in our modeling predictions with M-model 1 and in a previous experimental study (Larsen and Olesen, 2010). The reason is that the hERG1 b-isoform has both faster activation kinetic and faster recovery from inactivation kinetics that results in a net increase current compared to the hERG1 a-isoform.

## Conclusions

We investigated structural and biophysical properties of hERG1a and 1b homo-tetramers in the context of previously proposed Markov models and new data measured in the HEK cell line. Two M-models were tested and fitted to the experimental data. For the first time a set of parameters were provided for both isoforms. The models' parameters were then used to investigate effects of various homo-tetramers ratios formed by two isoforms in cardiac cells and tissue to track isoform-specific effects on emergent behaviors that occur in higher dimensions. The minimization procedure presented here, allowed assessment of suitability of different Markov model topologies and the corresponding parameters that describe the channel kinetics. In terms of the gating kinetics, we found that both M-models were able to qualitatively capture the kinetics of two isoforms. The kinetic modeling showed a profound difference in hERG1a and hERG1b deactivation rates, where the quantitatively fitted parameters to the data suggested that the hERG1b late deactivation rate is between 8 and 15 times faster than hERG1a.

In order to gain insight and link the observed isoforms' differences to the structure, full channel structural models were developed and analyzed for open and closed states. From the structural point of view, open and closed structural models for the full channel were for the first time compared providing hypothetical structural mechanism for transitions between closed to open states of hERG channel. In line with the kinetic modeling, interactions between soluble domains and the TM part of the channel appeared to be critical determinants of the gating kinetics allowing explanation of apparent differences in the deactivation rates between two isoforms. The model emphasized importance of the electrostatic interactions between N-cap of PAS domain and TM domain. To test the proposed role of stabilizing interactions between N-cap of PAS domain and the gating machinery in TM, we examined gating kinetics of E544L. Introduction of charge neutralizing resulted in significantly enhanced deactivation rates, reminiscent of isoform-specific differences. We attribute it to interactions between E544 and R681 missing in E544L mutant. Importantly, this interaction is present in both hERG1 a-isoform and hERG1 b-isoform, however b-isoform is missing the of PAS domain who might contribute to stabilize that interaction. While this work was under review, another publication by de la Peña et al. (2018) showed that hERG gating profiles can be reconsiled from non-covalently linked VSD and Pore Domain. Their findings, in line to what is presented in this work, challenge the classical view of the S4–S5 linker acting as lever to open the gate, supporting the hypothesis that the S4–S5 linker might integrate signals coming from the cytoplasmic domains (c-linker/PAS). Importantly, those split-channels disconnected at the S4–S5 linker show a destabilization of the closed state, in particular one of the split shown to be near E544 position discussed in our submission. Our structural modeling is providing a first structural glimpse of the structural underpinnings of the peculiar isoforms' gating and suggesting potential key interactions between S4–S5 linker, C-linker and PAS. Equally important question discussed in our study is the potential impact on the Action Potential from different ratios of isoform expression in the myocytes. The AP simulations performed in our study suggest that recovery from inactivation of hERG1 B may contribute to its physiologic role of b-isoform in the action potentials. Both structural and functional models were exploratory in nature aiming to provide a perspective for future multi-scale modeling studies.

In conclusion, the results and in-depth review of modeling, structural and functional data presented here contribute to the growing body of evidence that hERG1b significantly affects the generation of the cardiac I_kr_ and plays an important role in cardiac electrophysiology.

## Author contributions

JG: Performed all of the electrophysiological recordings and mutagenesis experiments, analyze results and wrote the manuscripts. LP and PD: Wrote the software; LP, ML, and P-CY: Performed simulations and analyzed the data; CC, SN, and HD: Supervised the research; LP, HD, CC, and SN: Designed the research. All authors wrote the article.

### Conflict of interest statement

The authors declare that the research was conducted in the absence of any commercial or financial relationships that could be construed as a potential conflict of interest.

## Footnotes

^1^Voltage-Gated Ion Channels Kinetic Modeling from whole-cell voltage-clamp data (VGC-KiMo) is a standalone tool written in C++ language, working on Linux machines and parallelized with OpenMP (see the Supporting Material for the scalability benchmarks). VGC-KiMo code is distributed under GNU General Public License thus freely available for download from https://github.com/vgckimo/vgckimo, together with documentation and tutorial files. See Supporting Material for a complete description of the code.
