# Supplementary material for: Determinants of Isoform-Specific Gating Kinetics of hERG1 Channel: Combined Experimental and Simulation Study
Source: Front Physiol. 2018 Apr 12;9:207. doi: 10.3389/fphys.2018.00207 (PMC5907531; doi:10.3389/fphys.2018.00207)
Supplement: Supplementary file 1 [file DataSheet1.PDF]

SUPPLEMENTAL MATERIALS

Determinants of Isoform-Specific Gating Kinetics of hERG1 Channel: Combined Experimental and Simulation Study.

Laura L. Perissinotti[1*,] Pablo M. De Biase[1], Jiqing Guo[2], Pei-Chi Yang[3],
Miranda C. Lee[1], Colleen E. Clancy[3*], Henry Duff[2*], and Sergei Y. Noskov[1,*]

[1]Centre for Molecular Simulations, Department of Biological Sciences, Faculty of Science, University of Calgary, Calgary, Alberta, Canada.

[2]Libin Cardiovascular Institute of Alberta, Faculty of Medicine, University of Calgary, Calgary, Alberta, Canada

[3]Department of Pharmacology, University of California at Davis, Davis, California, United States of America.

Submitted: Frontiers in Physiology
Special Issue: Safety Pharmacology - Risk Assessment QT Interval Prolongation and Beyond

LLP, PMDB, JQ AND PCY EQUALLY CONTRIBUTED

AUHTORS FOR CORRESPONDENCE:
LLP, CEC, HJD OR SYN

VGC-KiMo Code

1-Code Description and Features

This computational tool offers features that allow for different fitting strategies: initial values searching, around the initial guess or specified boundaries, minimization with fixed parameters and applying boundaries, and customizable weight for the individual terms in the cost function. A combination of these different fitting strategies is helpful for refining and improving the fitting and provides a useful tool to understand the channel gating kinetics and discriminate between different M-models. It provides electrophysiologists a useful computational tool to develop and optimize a computational model to gain insights into ion channel kinetics, test the effects of perturbations and, if desired, to incorporate ion channel models into higher dimensional models to simulate cellular and tissue dynamics effects. The current release includes voltage protocols to assess the effect of a drug, these protocols were recently included and future releases will extend their application.

VGC-KiMo source code is in C++. A flowchart for the parameters minimization procedure is depicted in Figure 3. VGC-KiMo is an interactive standalone program. It can be executed using an input file that answers all VGC-KiMo prompts or the end user can answer the prompts interactively. For every question, information between the brackets is the default option that can be used by answering each prompt with an empty line. Steps to follow through the optimization procedure:

1- *Initial Values:* It is possible to perform an initial search prior to optimization in case the initial values are inadequate or insufficient for optimization. Optimization convergence may be elusive when the algorithms dealing with many parameters get stuck in a local minimum. In this situation an initial values search may be helpful. The initial guess is done by generating biased random initial values for the optimizable parameters and selecting the parameter sets that provide the lowest total cost function. There are two available random number generators (RNG): rectangular (uniform) and Gaussian distribution.

2- *Voltage Clamp protocols:* VGC-KiMo includes the most commonly used voltage protocols (see supplemental materials). The total number of protocols needs to be specified and for each protocol, the external data file (experimental data) together with details about the protocol (number of steps, voltage magnitudes, duration) needs to be input.

3- *M-model for the current:* The M-model should be selected. All rate constants (k) have the form of eq. 3., where i refers to the transition rate between certain states (α has ms^{-1} units and β mV^{-1} units). The differential equation system from the M model is solved numerically by Runge-Kutta algorithm (1)(4th order, with a time step that should be specified).

Current VGC-KiMo includes two different Markov models for hERG K^+ (I_{Kr} current, Fig. 2). Any other model can be included in the source code in a separate function(2, 3).

$$k_i(V) = \alpha_i \; exp(\beta_i \; V) \; (1)$$

4- *Minimization procedure:* The cost function (***F***) is defined as the sum of weighted squared differences between experimental and simulated values for each voltage protocol (see eq. 2, n is the number of voltage protocols and eq. 3, l is the number of data points). ***F*** is then minimized using the PRAXIS algorithm(4) by varying the correction coefficients (a_i and b_i) of the initial kinetic parameters (eq. 4).

Iteration is converged at a given value of tolerance. The code allows the fixation of parameters and the application of boundaries.

$$F = \sum_{j=1}^{n} w_j f_j(x_1, \dots, x_m) \quad (2)$$

$$f_j(x_1, \dots, x_m) = \frac{1}{l} \sum_{h=1}^{l} \left(y_h - y_h^{exp}\right)^2 \quad (3)$$

$$k_i(V) = a_i \alpha_i \; exp\left(b_i \beta_i \; V\right) \quad (4)$$

5- *Convergence Analysis:* VGC-KiMo can perform a convergence analysis on the cost function (F)(5, 6) and all partial cost functions (f_j) (*eq. 2,3*). The analysis consists in the numerical computation of the first and second derivatives of F and f_j as a function of all adjusted parameters $(x_1, \dots, x_m)$. The procedure requires the computation of a vector and a matrix. The vector is constructed by computing all the cost functions while varying by 0.01 each parameter around the optimized value. In the same way, the matrix is built by changing two parameters at a time. Subsequently, a system of linear equations is solved to obtain the parameters of a second order Taylor polynomial of several variables. The first derivative terms account for the convergence of the cost function for each parameter. The self-second derivative terms provides information about the sensitivity of the cost functions to each parameter. The cross-second derivative terms account for the dependence of pairwise parameters and can be used to distinguish redundant parameters. All these terms together provides information about the completeness of the experimental data set to be unambiguously described by the kinetic parameters of the proposed test mechanism.

6- *Final optimized parameters:* output files with the simulated data after the fitting are generated for each protocol included.

7- *Single point cost function evaluation:* VGC-KiMo can also be used to simulate data for given a set of parameters without any optimization procedure.

2-Validation : Kv11.1 Kinetics

2.1- Parameters from literature

Table S1. Published parameters for diferent M-models (see Fig. 2) and cell lines at room temperature (rt).

Transition	label	**α** (*ms*$^{-1}$)			*β* (*mV*$^{-1}$)		
		WLMSR		MGWMN(2)	WLMSR		MGWMN
		Fink et al.(7) hEK, rt	Rassmuson(3) oocytes, rt	hEK, rt	Fink et al. hEK, rt	Rassmuson oocytes, rt	hEK, rt
C3-->C2	*ae*	5.78E-2	2.23E-2	6.9E-3	0.0117	0.0117	0.0272
C2-->C3	*be*	6.03E-2	4.70E-2	2.27E-2	-0.0631	-0.631	-0.0431
C2-->C1	*ain*	1.18E-2	2.37E-2	2.66E-2	0	0	0
C1-->C2	*bin*	3.68E-2	3.67E-2	1.35E-1	0	0	0
C1-->O	*aa*	6.17E-8	1.37E-2	2.18E-2	0.0382	0.0382	0.0262

O-->C1	*bb*	1.03E-4	6.89E-5	9.0E-4	-0.0418	-0.0418	-0.0269
O-->I	*bi*	9.26E-2	9.08E-2	6.22E-2	0.0233	0.0234	0.0120
I-->O	*ai*	8.28E-3	6.49E-3	5.9E-3	-0.0327	-0.0327	-0.0443
C1->I	*bi**	-----	-----	1.29E-5	-----	-----	2.71E-6

Table S2. Performance of different optimization algorithms for derivation of WT hERG/hEK values at 23 C: Nelder Mead Simplex (Our implementation : NMS/ Matlab) and Praxis (VGCKiMo) hEK data (8).

Transition	label	**α** (ms^{-1})			*β* (mV^{-1})		
		Fink et al. (NMS)	NMS	Praxis	Fink et al.	NMS	Praxis
C3-->C2	*ae*	5.78E-2	5.78 E-2	8.67E-2	0.0117	0.0117	0.0212
C2-->C3	*be*	6.03E-2	6.01 E-2	3.60E-2	-0.0631	-0.0631	-0.0734
C2-->C1	*ain*	1.18E-2	9.22 E-3	1.02E-2	0	0	0
C1-->C2	*bin*	3.68E-2	3.65 E-2	2.99E-2	0	0	0
C1-->O	*aa*	6.17E-8	6.21 E-3	1.16E-2	0.0382	0.0382	0.0438
O-->C1	*bb*	1.03E-4	1.36 E-4	1.36E-4	-0.0418	-0.0418	-0.0418
O-->I	*bi*	9.26E-2	1.25 E-1	1.25E-1	0.0233	0.0233	0.0231
I-->O	*ai*	8.28E-3	3.64 E-3	3.64E-3	-0.0327	-0.0327	-0.0327

2.2- Rate Formulations & Model Fitting to CHO Data –Isoform a-

All rate constants were written according to eq 1. The transitions between O and I states (see Fig. 2) are also dependent on extracellular potassium concentration $[K^+]^0$ (9). That dependence is accounted for in moldel M 1 (Fink et al. (7)) by modifying the transition rate for inactivation ($O \rightarrow I$, *bi*) as stated in eq. 5.

$$k_{bi}([K^+]^0) = k'_{bi}\left({5.4mM}/{[K^+]^0}\right)^{0.4} \quad (5)$$

2.2.1- Deactivation : Experimental data for CHO at room temperature.

Table S3. Deactivation parameters from exponential fitting to experimental CHO data, data extracted from Fig.5 (Larsen et al. (10)).

Voltage(mV)	τ_{fast} (ms)	τ_{slow} (ms)	$A_{fast}/(A_{fast}+A_{slow})$
Isoform a			
-100	25.0	210.0	0.90
-60	133.7	666.6	0.42
-50	187.8	1170.0	0.3
Isoform b			
-120	6	46	0.99
-60	32	160	0.99
-50	43	197	0.95

2.2.2-Experimental voltage clamp protocols for the fitting of WT-hERG at room temperature

Voltage protocols

Protocol 0 (Steady State Activation: SSA) (11): Holding potential was set to -80 mV (V1, V2), activation of hERG was induced by depolarization to a range of potential from +60 to -90 mV (V3) in 10-mV steps for 1s. This was followed by re polarization at -60 mV (V4) for 500 ms to record tail currents. The normalized peak tail currents meassured at -60 mV were plotted against the membrane potential of the previous voltage step

Protocol 0 (Steady State Inactivation: SSI) (SSI): Holding potential was set to -80 mV (V1), three step protocol was applied to investigate voltage dependent recovery from inactivation. From a potential of +40mV (V2), the channels were subjected to brief (10 ms) hyperpolarizations from +40 to -120 mV (V3) in 10 mV decrements . The peak current amplitude after return to +40 mV (V4) was normalized and plotted against the potential of the hyperpolarizing step.

Protocol 1 (Envelope of tails: Activation) (ACT): Holding potential was set to -80 mV (V1). The protocol was carried out by activating channels at +40 mV (V2) for various durations of time (5-500 ms) and then measuring the tail current at -60 mV (V3). The peak amplitude of the tail current were normalized to the maximun amplitude and plotted as a function of the duration of the activating step.

Protocol 2 (Deactivation at -100 mV and V0.3): Holding potential was set to -80 mv(V1), the channel was first depolarized to +40 mV (V2) for 1000 ms, followed by step potential from -120 to + 50 mV (V3) for 4 s. The deactivating current traces were best fitted to a double exponential function.

2.2.3-Fitted parameters for Isoform a

Table S4. Correction factors (eq. 1) applied to the original models (see Table S1 for the ***α*** (*ms^{-1}*) and ***β*** (*mV^{-1}*) values) after fitting the CHO data to M-model 1 and M-model 2.

Transition	label	Boundaries	M-model 1		M-model 2	
			**a**	*b*	**a**	*b*
C3-->C2	*ae*	0.8-2	0.80	0.80	0.80	1.26
C2-->C3	*be*	0.8-2	0.93	1.05	0.89	0.99
C2-->C1	*ain*	0.8-2	1.69	---	1.10	---
C1-->C2	*bin*	0.8-2	0.80	---	0.91	
C1-->O	*aa*	0.1-10	0.68	1.27	1.14	1.25
O-->C1	*bb*	0.1-10	6.97	0.71	0.94	0.81
O-->I	*bi*	0.8-2	1.99	0.95	1.42	1.46
I-->O	*ai*	0.8-2	0.80	0.89	1.14	1.24
C1->I	*bi**	0.1-1000	---	---	0.1	4.12
F			8.35 e-4		5.81e-4	

Table S5. Correction factors (eq. 2) applied to the original models after doing additional fitting the CHO data to M-model 1 and M-model 2.

Transition	label	Boundaries M1/M2	M-model 1(M1)		M-model 2(M2)	
			**a**	***b***	**a**	***b***
C3-->C2	*ae*	0.7-2/0.8-2	0.71	0.76	0.80	1.04
C2-->C3	*be*	0.7-2/0.8-2	0.81	1.1	0.95	0.99
C2-->C1	*ain*	0.7-2/0.8-2	1.76	---	1.36	---
C1-->C2	*bin*	0.7-2/0.8-2	0.73	---	0.86	
C1-->O	*aa*	0.1-10	0.62	1.29	0.93	1.39
O-->C1	*bb*	0.1-10	7.27	0.72	1.18	0.56
O-->I	*bi*	0.7-2/0.8-2	1.99	0.89	1.13	1.42

I-->O	*ai*	0.7-2/0.8-2	0.70	0.94	0.80	0.80
C1->I	*bi**	0.1-1000	---	---	0.62	4.12
F			5.25 e-4		3.61e-4	

3-Software scalability and benchmark

The code was parallelized using OpenMP. Implementations were run on HP Xeon E5649 cluster that consists of 12-core compute nodes. The 12 cores associated with one node share 24GB of RAM. The efficiency of parallelization for 4 processors is close to 80% and starts to decay above 4. The code was compiled using Intel compilers activating O3 optimizations and the highest instruction set available at the host processor.

Table S6. Benchmark for M-model 2, 16 parameters in total, 4 voltage protocols. Speed up(C)=ET(1)/ET(C); Eficiency=Speedup(C)/C.

Cores (C)	Elapsed Time (ET:minutes)	Speed up	Efficiency (%)
Test6 no random generator (F_{ini}:5.20E-2, F_{end}:5.81E-4)			
1	1830	-	100
4	580	3.16	79
8	420	4.36	54.5
12	405	4.52	37.7

Table S7. Benchmark for M-model 2, 16 parameters in total, 4 voltage protocols. Speed up(random)=ET(No random)/ET(random). All tests were done for four -cores runs submitted in computer cluster. For all runs, sampling of initial guesses was set to 1000 tries.

Test6 (4 C)	Elapsed time *(ET:minutes)	Speed up	F_{ini}	F_{end}
No random	580	-	5.20E-2	5.81E-4
random1	310(30)	1.87	6.26E-4	5.48E-4
random2	280(30)	2.07	1.37E-3	5.21E-4
random3	360(60)	1.62	1.47E-3	6.01E-4

*Optimization time + Sampling time (Sampling time : time spent searching for initial guess)

Table S8. Performance and benchmark for different number of voltage protocols.

Number of Protocols	type of Protocol	Elapsed Time (hours)	Individual Cost Function (fx)
1	0	6:00	f0:1.63E-7
1	4	0:15	f4:2.34E-4
2	0,4	4:00	f0:1.67E-6, f4:1.83E-4
3	0,1,4	5:00	f0:1.05E-4, f1:1.67E-4, f4:1.82E-4
4	0,0,1,4	9:50	f0:1.14E-4, f0:9.01E-5, f1:1.72E-4, f4:2.042E-4

4- Rate Formulations & Model Fitting to CHO Data : Isoform a vs b

Electrophysiological data using the CHO cell line was obtained from Larsen, A.P., et al.

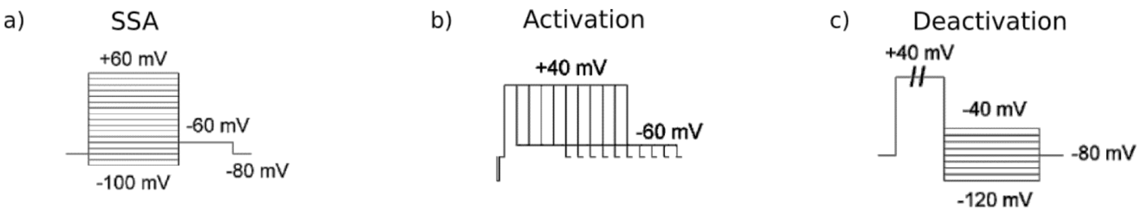

Electrophysiological voltage protocols considered. The three protocols used for fitting rate constants for a) steady state activation (11), b) activation (envelope of tails), and c) deactivation kinetics at -100, -50 and -60 mV. No significative changes were meassured in inactivation rates. Small change in recovery from inactivation.

Table S9. M-model 1 Rate Constants for Transitions within hERG Gating for a-isoform and b-isoform. CHO cells data at room temperature.

Transition	Parameter name		Rates		ratio b/a
			Isoform a -CHO-	Isoform b –CHO-	
C3→C2	ae	α	0.0693639882	0.0693625143	**1.00**
		β	0.028355636	0.019088162	0.70
C2→C3	be	α	0.03654	0.06829344	**1.87**
		β	-0.07349909	-0.066070276	0.90
C2→C1	ain	α	0.015878952	0.018906006	1.20
C1→C2	bin	α	0.0239202392	0.0246606529	1.03
I→O	ai	α	0.0039120536	0.0113719788	**2.90**
		β	-0.0324997057	-0.0324427887	1.00
O→I	bi	α	0.1010555	0.124884625	1.24
		β	0.041966232	0.023307438	0.56
C1→O	aa	α	0.0109215392	0.049263576	**4.51**
		β	0.05935995	0.0394235916	0.70
O→C1	bb	α	0.00016612808	0.001549584	**9.33**
		β	-0.044634458	-0.063257194	1.42

Where α (1/ms) indicates voltage independant rate, and β (1/mV) indicates voltage dependant rate as follows: k= α*exp(β*V) The transitions between O and I states are also dependent on extracellular potassium concentration $[K^+]^0$. That dependence is accounted for in moldel by modifying the transition rate for inactivation ($O \rightarrow I$, bi) as: $k_{bi}([K^+]^0)=k'_{bi}(5.4mM/[K^+]^0)^{0.4}$

Table S10. Rate Constants factor corrections for Transitions within hERG Gating M-model 2 for isoform a and isoform b. CHO data at room temperature.

Parameter Name		MGWMN (ms-1)	Correction factors	
			Isoa (CHO)	isob(CHO)
ae	α	0.0069	0.800032	1.07577
	β	0.0272	1.21655	0.858628
be	α	0.0227	1.08597	0.800571
	β	-0.0431	1.08559	0.800093
ain	α	0.0266	1.146	1.32593
bin	α	0.1348	0.834551	0.855167
ai	α	0.0059	0.832631	2.21861 **(2.66 times)**
	β	-0.0443	0.872266	0.986773
bi	α	0.0622	1.43517	0.900581
	β	0.0120	0.809065	0.900017
aa	α	0.0218	1.16426	5.11452 **(4.39 times)**
	β	0.0262	1.1962	0.812985
bb	α	0.0009	0.775023	10.4073 **(13.43 times)**
	β	-0.0269	0.871463	0.992474 (1.14 times)
bii	α	1.29e-5	100.558	0.100008
	β	2.71e-6	98.698	8.44802

5-Alternative Fits to hEK cell data

An alternative good fit of M-model 1 to the experimental data is provided in Table S11. Note that the main difference is located in the late deactivation rate (much faster) and recovery from inactivation (invariant). These parameters were tested

in AP simulation and they were not able to capture the short APD of b-isoform compared to a-isoform. This result added an additional level of validation for the Fit2 set of parameters presented and discussed in the manuscript.

Table S11. M-model 1 Rate Constants for Transitions within hERG Gating for a-isoform and b-isoform. Fit 2 corresponds to the parameters showed in the manuscript and Fit 1 is an alternative fit.

Transition	Parameter name		Rates			ratio b/a	
			b-Isoform		a-Isoform		
			Fit1	Fit2		Fit1	Fit2
C3→C2	ae	α	3.73E-2	4.71 E-2	3.39 E-2	1.10	**1.39**
		β	7.23 E-3	9.36 E-3	1.04 E-2	0.70	0.90
C2→C3	be	α	4.33 E-2	7.43 E-2	4.82 E-2	0.90	**1.54**
		β	-5.11 E-2	-5.05 E-2	-6.91 E-2	0.74	0.73
C2→C1	ain	α	4.54 E-2	5.01 E-2	2.20 E-2	**2.07**	**2.28**
C1→C2	bin	α	6.94 E-3	2.95 E-2	1.36 E-2	**0.51**	**2.17**
I→O	**ai**	α	7.54 E-3	2.80 E-2	6.63 E-3	1.14	**4.23**
		β	-4.01 E-2	-3.06 E-2	-3.89 E-2	1.03	0.79
O→I	bi	α	8.37 E-2	7.41 E-2	7.41 E-2	1.13	1.00
		β	1.30 E-2	1.88 E-2	2.80 E-2	0.46	0.67
C1→O	**aa**	α	1.36 E-2	1.71 E-2	4.94 E-3	**2.75**	**3.46**
		β	3.61 E-2	3.05 E-2	4.31 E-2	0.84	0.71
O→C1	**bb**	α	1.17 E-2	1.61 E-3	2.06 E-4	**57.02**	***7.83***
		β	-1.71 E-2	-3.44 E-2	-3.76 E-2	0.46	0.91

6-Physiological Action Potential simulations

Table S12. Physiological action potential simulations were subsequently performed at 37°C. b-Isoform (second column) and a-Isoform (third column) transition rate constants. T_{base} corresponds to 310 K (12).

	b-Isoform	a-Isoform

C_3 → C_2	$ae = \frac{T}{T_{base}} 4.71\times 10^{-2} \times e^{(\frac{T_{base}}{T}\times 9.36\times10^{-3}\times V)}$	$ae = \frac{T}{T_{base}} 3.39\times 10^{-2} \times e^{(\frac{T_{base}}{T}\times 1.04\times10^{-2}\times V)}$
C_2 → C_3	$be = \frac{T}{T_{base}} 7.43\times 10^{-2} \times e^{(\frac{T_{base}}{T}\times -5.05\times10^{-2}\times V)}$	$be = \frac{T}{T_{base}} 4.82\times 10^{-2} \times e^{(\frac{T_{base}}{T}\times -6.91\times10^{-2}\times V)}$
C_2 → C_1	$ain = \frac{T}{T_{base}} 0.05$	$ain = \frac{T}{T_{base}} 0.022$
C_1 → C_2	$bin = \frac{T}{T_{base}} 0.0295$	$bin = \frac{T}{T_{base}} 0.0136$
C_1 → O	$aa = \frac{T}{T_{base}} 1.71\times 10^{-2} \times e^{(\frac{T_{base}}{T}\times 3.05\times10^{-2}\times V)}$	$aa = \frac{T}{T_{base}} 4.94\times 10^{-3} \times e^{(\frac{T_{base}}{T}\times 4.31\times10^{-2}\times V)}$
O → C_1	$bb = \frac{T}{T_{base}} 1.61\times 10^{-3} \times e^{(\frac{T_{base}}{T}\times -3.44\times10^{-2}\times V)}$	$bb = \frac{T}{T_{base}} 2.06\times 10^{-4} \times e^{(\frac{T_{base}}{T}\times -3.76\times10^{-2}\times V)}$
O → I	$bi = \frac{T}{T_{base}} 7.41\times 10^{-2} \times e^{(\frac{T_{base}}{T}\times 1.88\times10^{-2}\times V)} \times \left(\frac{5.4}{[K]^o}\right)^{0.4}$	$bi = \frac{T}{T_{base}} 7.41\times 10^{-2} \times e^{(\frac{T_{base}}{T}\times 2.8\times10^{-2}\times V)} \times \left(\frac{5.4}{[K]^o}\right)^{0.4}$
I → O	$ai = \frac{T}{T_{base}} 2.8\times 10^{-2} \times e^{(\frac{T_{base}}{T}\times -3.06\times10^{-2}\times V)}$	$ai = \frac{T}{T_{base}} 6.63\times 10^{-3} \times e^{(\frac{T_{base}}{T}\times -3.89\times10^{-2}\times V)}$

7-Deactivation : Experimental data for hEK cells at room temperature.

Table S13. Experimental data for HEK cells at room temperature. Each value is an average of n experiments. Deactivation time course at each voltage was fitted to double exponential function: A_{fast}exp(-x/τ_{fast})+A_{slow}(-x/ τ_{slow}), see Table S14.

	hERG1a	hERG1b
**Deactivation**		
τ_{fast} (ms)	(n=10)	(n=10)
-40 mV	41.5 ±2.9	10.5 ±1.1
-60 mV	64.4 ±5.3	6.1 ±1.1
-100 mV	69.6 ±2.1	11.5 ±1.6
-120 mV	39.9 ±2.3	10.6 ±2.2
τ_{slow} (ms)		
-40 mV	878.9 ±33.9	197.7 ±13.2
-60 mV	780.5 ±27.9	52.9 ±2.4
-100 mV	329.3 ±13.1	45.7 ±1.9
	492.6 ±35.2	-------

-120 mV		
$A_{fast}/A_{slow}+A_{fast}$		
-40 mV	0.42	0.70
-60 mV	0.31	0.55
-100 mV	0.78	0.77
-120 mV	0.98	1

The two exponential fittings show no apparent dependence of the fast component with voltage. In our case the fittings were done to the whole set of data (10 experiments), data were combined, after base line adjustments, then were averaged and then fitted. The values show a correlation with voltage when looking at the slow component manly, the components' relative contribution agrees to what has been observed before for CHO cells (Table S3).

It is important to emphasize that in our work we didn't used these times constants values in the model fitting procedure to avoid artifacts from fitting the data to two exponential functions, we just fitted the model to the average traces at each voltage. However, we used previously published times constants meassured for CHO (see section 2.2.1, table S9 and Fig. S5). Those constants, fast and slow agreed to was published before. Note that the fitted model parameters from CHO and our data share good similarities. It is important to mention that the kinetic model fitted poorly to the two exponentials CHO data, the "Afast/(Afast+Aslow)" ratio couldn't be reproduced by the model and mostly one exponential was fitted.

As a final note, Fink et. al. (Fink et al. (7)) mentioned and discussed some problems regarding inconsistencies between the model we are using and decativation times constants coming from experimental meassurements.

FIGURES

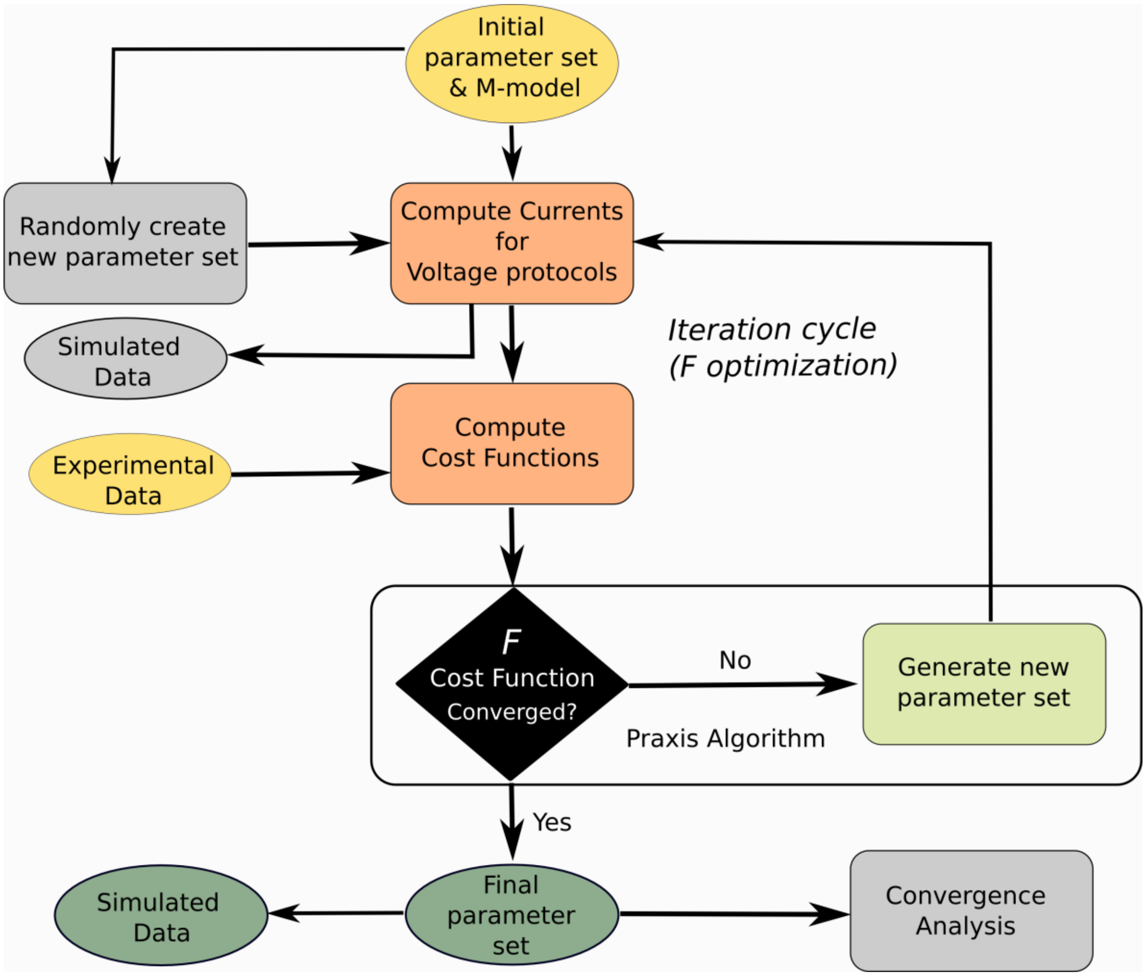

Figure S1. Flowchart in VGC-KiMo. Information read from the input file is in yellow ovals, output data is in green ovals. Currents for each voltage protocol are computed using the selected M-model. Tasks highlighted in grey are optional.

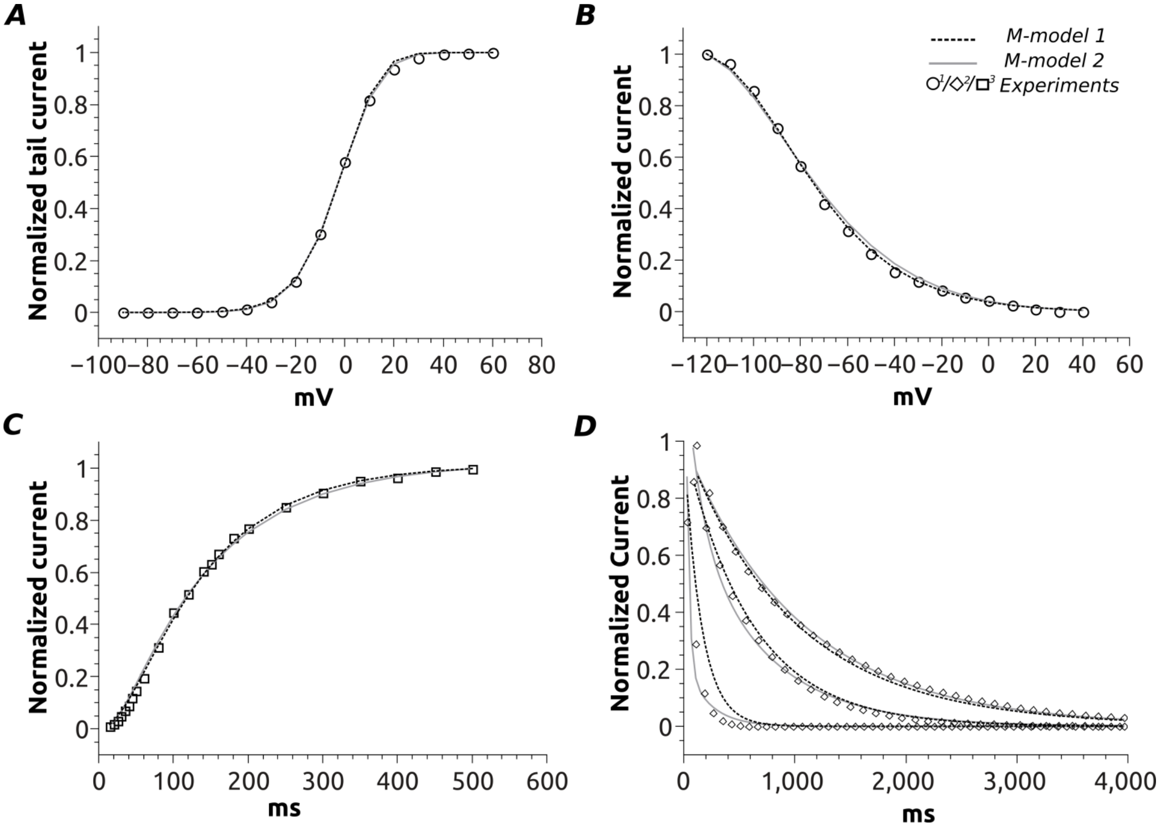

Figure S2. Simulated data using parameters derived from the fitting procedure to M-models 1 and 2. Experimental data on CHO cell line at room temperature(10). (A) Activation: $V_{1/2}$=-2.8±1.6 mV, slope=8.6.5±0.2 mV. (B) Inactivation:$V_{1/2}$=-82.0±3.0 mV, slope=-20.5±0.5 mV. [1]Data generated from Boltzmann function fit to experimental data. (C) Activation curve: [3] Experimental data extracted from Fig. 4, Larsen et al. (10). (D) Deactivation curves: [2]Data generated from exponential fit to experimental data, $\tau_{fast;}$ τ_{slow} in ms at -120, -60 and -50 mV (Table S8).

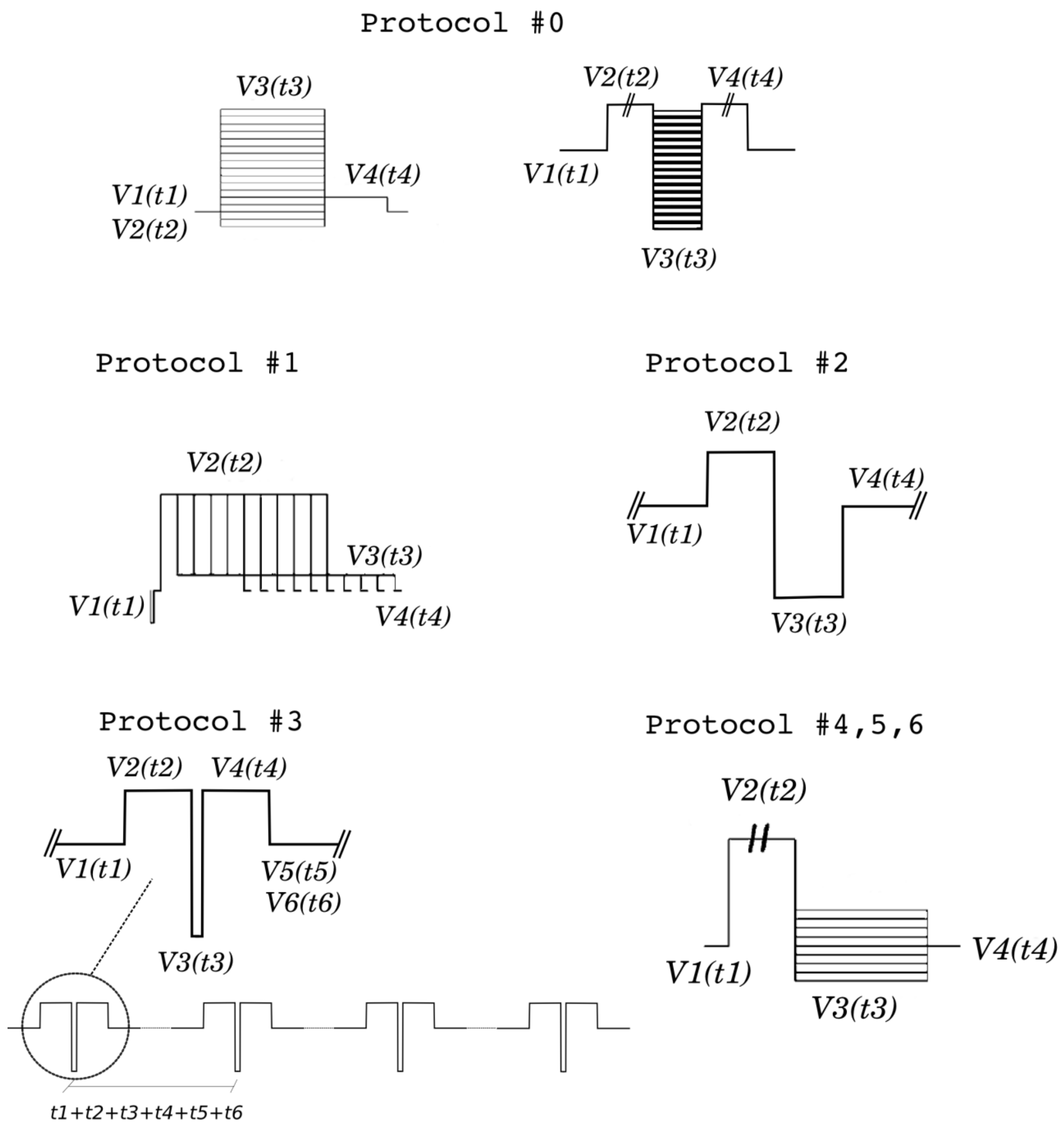

Figure S3. Voltage protocols available in the code.

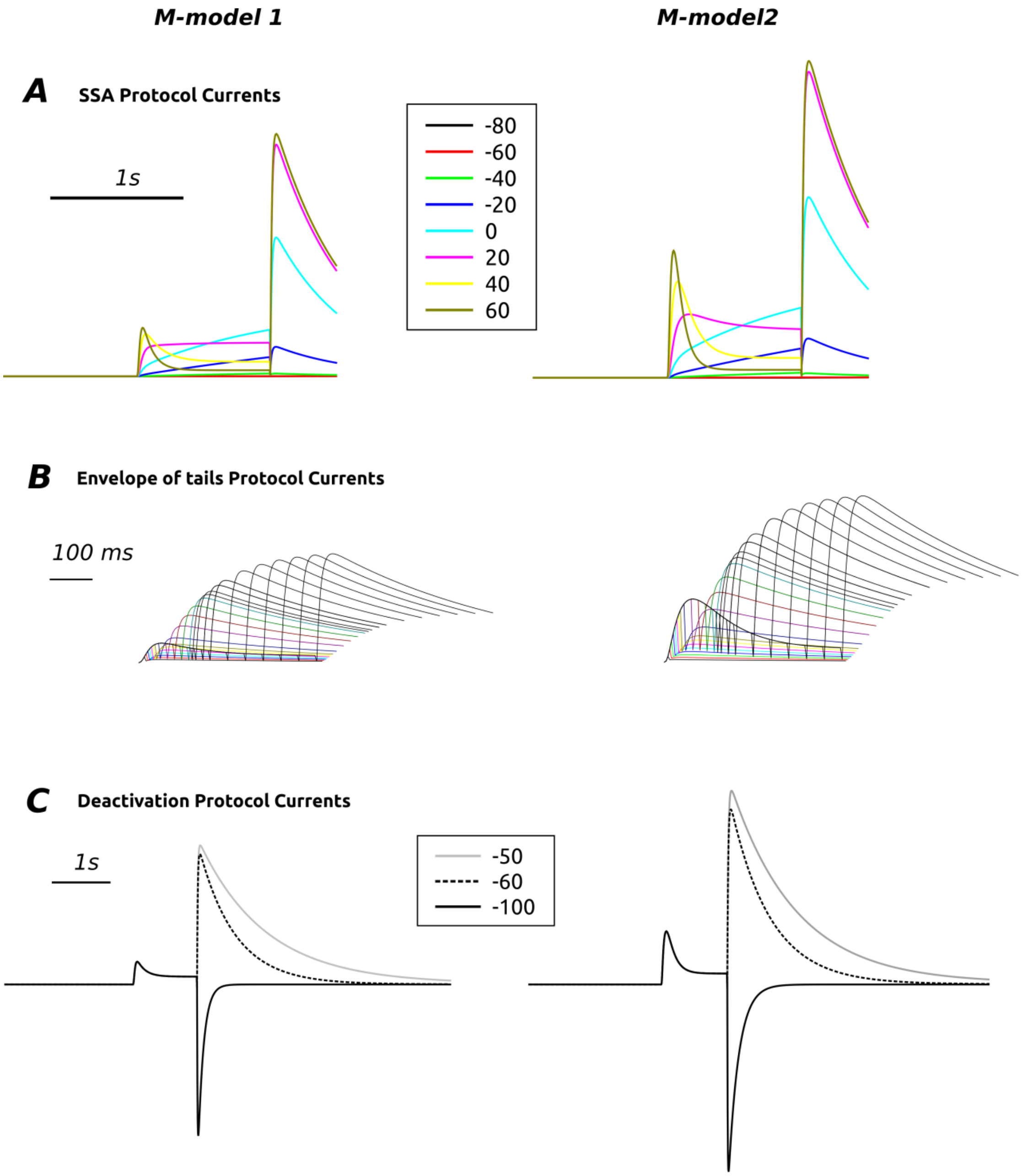

Figure S4. Simulated current traces elicited by voltage protocols 0 (A) and 1(B) and 4 (C) for M-models 1 and 2. Parameters correspond to fit shown in Table 1.

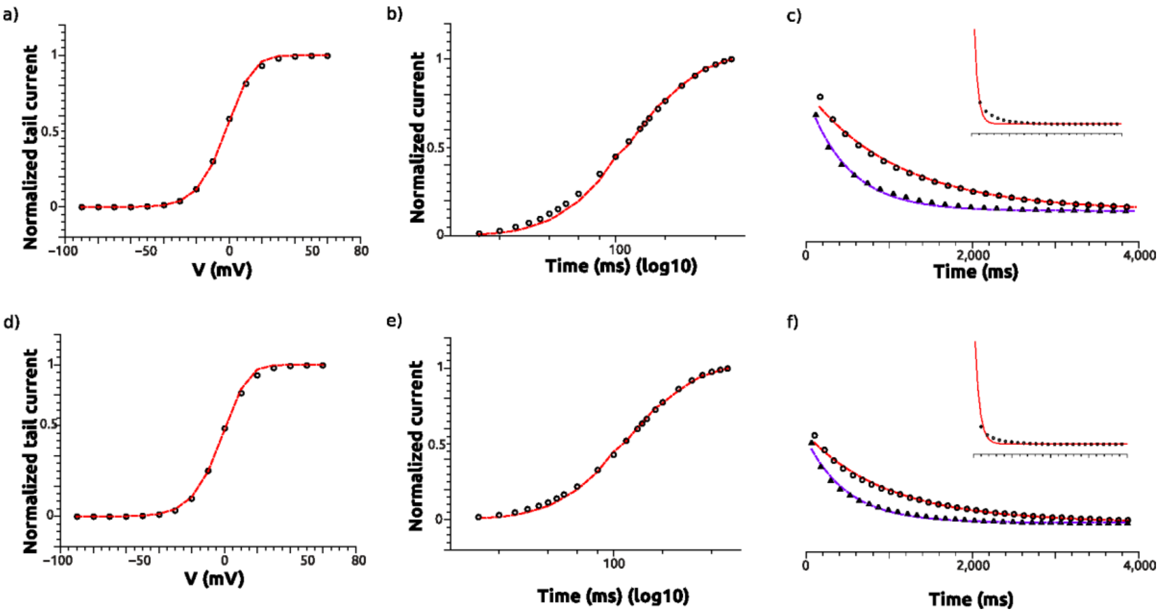

Figure S5. Simulated M-model 1 and 2 data compared to experimental data for isoform a -CHO cells-. Graphs a, b, and c are for mechanism 1 while graphs d, e and f are for mechanism 2. Graphs a and d correspond to the SSA protocol, graphs b and e correspond to the activation protocol, and graphs c and f correspond to the deactivation protocol. In graphs c and f, the red curve corresponds to a deactivation meassured at -50mV, the red curve corresponds to a deactivation at -60mV and the smaller deactivation graph corresponds to a deactivation at -100mV. In c and f current intensity is plotted against time. In all graphs the points correspond to experimental data while the dashed lines correspond to simulated data. All voltage protocols were performed as described in the methods section.

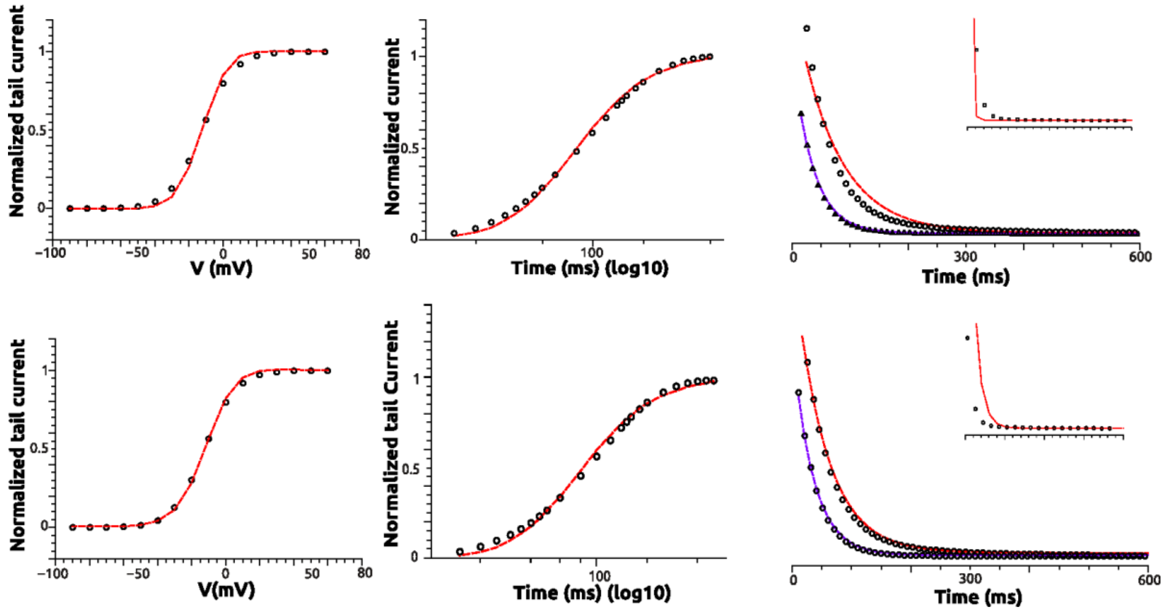

Figure S6. Simulated M-model 1 and 2 data compared to experimental data for isoform b -CHO cells-. Graphs a, b, and c are for mechanism 1 while graphs d, e and f are for mechanism 2. Graphs a and d correspond to the SSA protocol, graphs b and e correspond to the activation protocol, and graphs c and f correspond to the deactivation protocol. In graphs c and f, the red curve corresponds to a deactivation meassured at -50mV, the red curve corresponds to a deactivation at -60mV and the smaller deactivation graph corresponds to a deactivation at -120mV. In c and f current intensity is plotted against

time. In all graphs the points correspond to experimental data while the dashed lines correspond to simulated data. All voltage protocols were performed as described in the methods section.

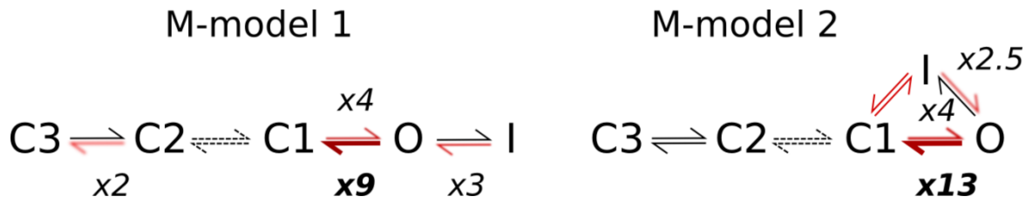

Figure S7. Relative rate changes for isoform b with respect to isform a fo CHO cells. See Tables S9 and S10.

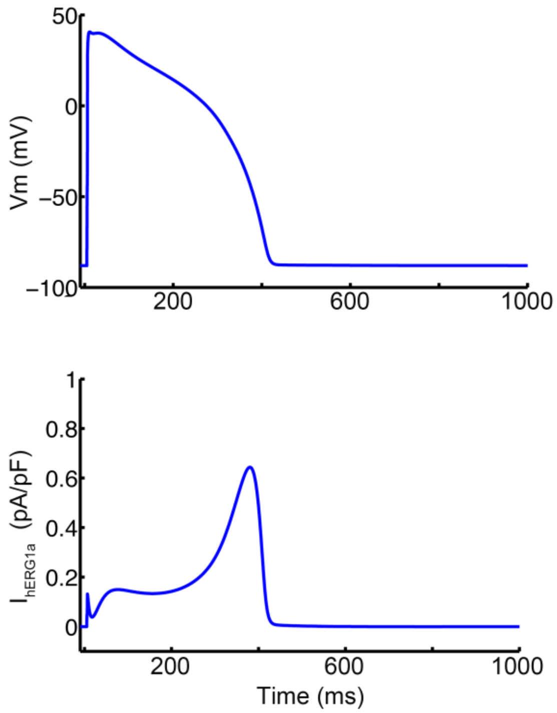

Figure S8. AP and Ikr for hERG1a. M-model1, parameters from Table 2.

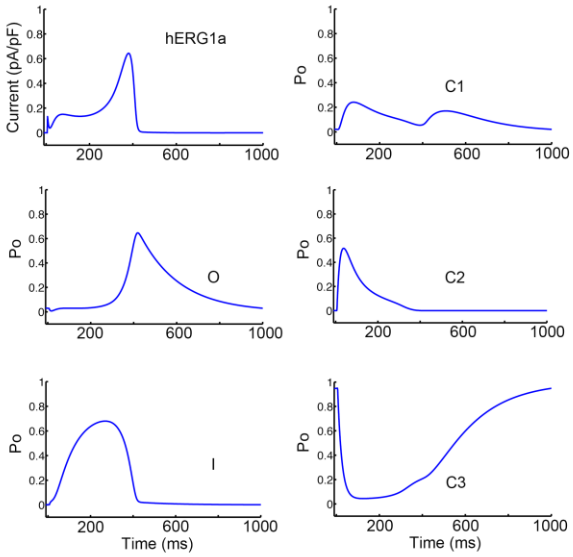

Figure S9. State occupancy during the AP for hER1a. M-model1, parameters from Table 2.

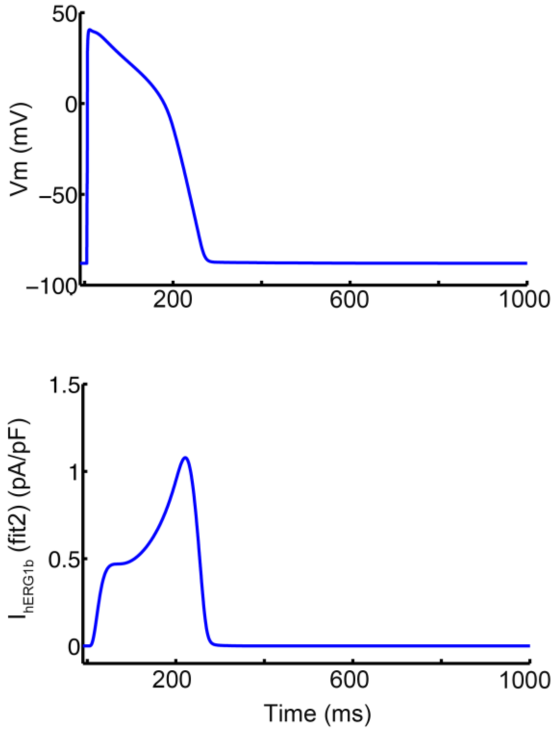

Figure S10. AP and Ikr for hERG1b. M-model1, parameters from Table 2.

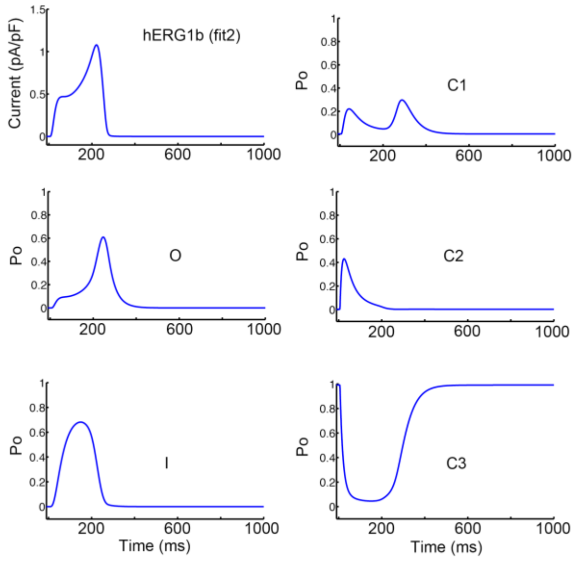

Figure S11. State occupancy during the AP for hER1b. M-model1, parameters from Table 2

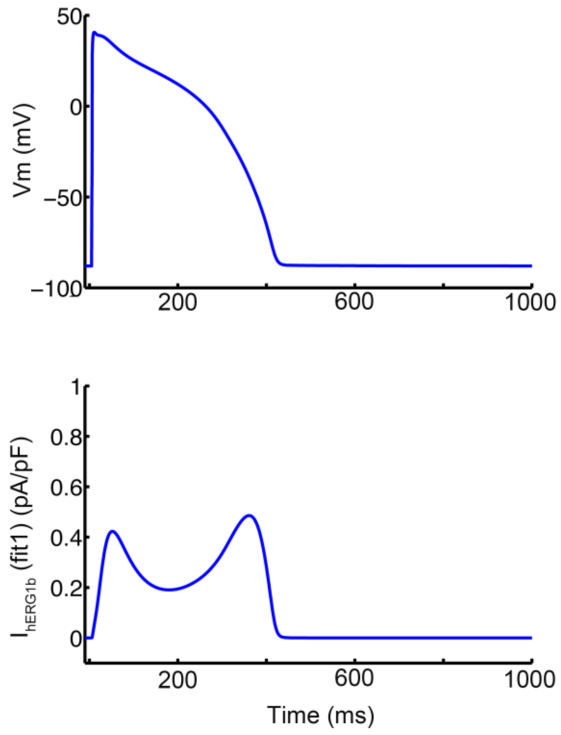

Figure S12. AP and Ikr for hERG1b. M-model1, parameters from Table S9.

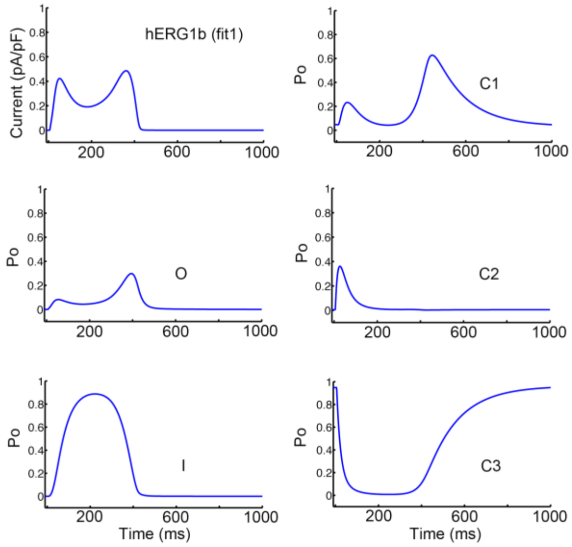

Figure S13. State Occupancy during AP for hERG1b. M-model1, parameters from Table S9.

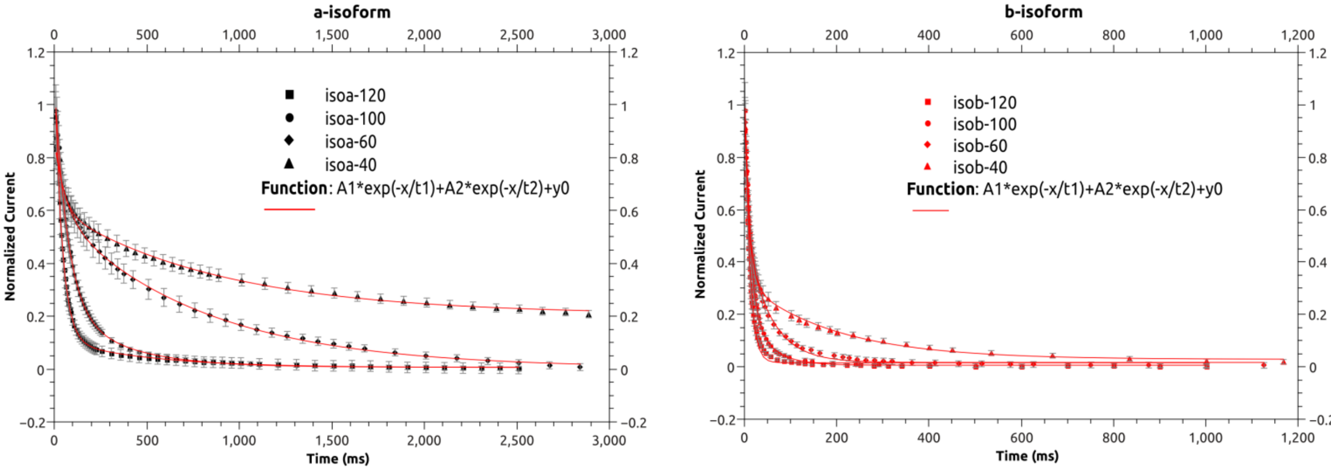

Figure S14. Deactivation experimental traces at different voltages. Symbols represent average value of n (n=10) traces with the corresponding error bar. Lines represent a double exponential fit to the experimental data. Parameters derived from the fit are in Table 1.

References

1. Press, W. H. F., Brian P.; Teukolsky, Saul A.; Vetterling, William T. 2007. Numerical Recipes: The Art of Scientific Computing Cambridge University Press.
2. Mazhari, R., J. L. Greenstein, R. L. Winslow, E. Marban, and H. B. Nuss. 2001. Molecular interactions between two long-QT syndrome gene products, HERG and KCNE2, rationalized by in vitro and in silico analysis. Circulation Research 89:33-38.
3. Wang, S. M., S. G. Liu, M. J. Morales, H. C. Strauss, and R. L. Rasmusson. 1997. A quantitative analysis of the activation and inactivation kinetics of HERG expressed in Xenopus oocytes. Journal of Physiology-London 502:45-60.
4. Brent, R. 1973. Algorithms for Minimization without Derivatives. Prentice Hall.
5. Turanyi, T. 1990. Sensitivity Analysis of Complex Kinetic Systems - Tools and Applications. Journal of Mathematical Chemistry 5:203-248.
6. Varga, L., B. Szabo, I. G. Zsely, A. Zempleni, and T. Turanyi. 2011. Numerical investigation of the uncertainty of Arrhenius parameters. Journal of Mathematical Chemistry 49:1798-1809.
7. Fink, M., D. Noble, L. Virag, A. Varro, and W. R. Giles. 2008. Contributions of HERG K+ current to repolarization of the human ventricular action potential. Progress in Biophysics & Molecular Biology 96:357-376.
8. Perissinotti, L. L., J. Guo, P. M. De Biase, C. E. Clancy, H. J. Duff, and S. Y. Noskov. 2015. Kinetic Model for NS1643 Drug Activation of WT and L529I Variants of Kv11.1 (hERG1) Potassium Channel. Biophysical Journal 108:1414-1424.
9. Yang, T., D. J. Snyders, and D. M. Roden. 1997. Rapid inactivation determines the rectification and [K+](o) dependence of the rapid component of the delayed rectifier K+ current in cardiac cells. Circulation Research 80:782-789.
10. Larsen, A. P., S.-P. Olesen, M. Grunnet, and T. Jespersen. 2008. Characterization of hERG1a and hERG1b potassium channels - a possible role for hERG1b in the I(Kr) current. Pflugers Archiv-European Journal of Physiology 456:1137-1148.
11. Crotti, L., D. J. Tester, W. M. White, D. C. Bartos, R. Insolia, A. Besana, J. D. Kunic, M. L. Will, E. J. Velasco, J. J. Bair, A. Ghidoni, I. Cetin, D. L. Van Dyke, M. J. Wick, B. Brost, B. P. Delisle, F. Facchinetti, A. L. George, Jr., P. J. Schwartz, and M. J. Ackerman. 2013. Long QT Syndrome-Associated Mutations in Intrauterine Fetal Death. Jama-Journal of the American Medical Association 309:1473-1482.
12. Romero, L., B. Trenor, P.-C. Yang, J. Saiz, and C. E. Clancy. 2015. In silico screening of the impact of hERG channel kinetic abnormalities on channel block and susceptibility to acquired long QT syndrome. Journal of Molecular and Cellular Cardiology 87:271-282.
